# Genes to therapy: a comprehensive literature review of whole-exome sequencing in neurology and neurosurgery

**DOI:** 10.1186/s40001-024-02063-4

**Published:** 2024-11-10

**Authors:** Joecelyn Kirani Tan, Wireko Andrew Awuah, Arjun Ahluwalia, Vivek Sanker, Adam Ben-Jaafar, Pearl Ohenewaa Tenkorang, Nicholas Aderinto, Aashna Mehta, Kwadwo Darko, Muhammad Hamza Shah, Sakshi Roy, Toufik Abdul-Rahman, Oday Atallah

**Affiliations:** 1 Faculty of Biology, Medicine and Health, The University of Manchester, Manchester, United Kingdom; 2 Faculty of Medicine, Sumy State University, Sumy, 40007 Ukraine; 3 School of Medicine, Queen’s University Belfast, Belfast, UK; 4 Department of Neurosurgery, Trivandrum Medical College, Thiruvananthapuram, India; 5 University College Dublin, School of Medicine, Belfield, Dublin 4, Ireland; 6 University of Ghana Medical School, Accra, Ghana; 7 Internal Medicine Department, LAUTECH Teaching Hospital, Ogbomoso, Nigeria; 8 University of Debrecen-Faculty of Medicine, Debrecen, Hungary; 9 Department of Neurosurgery, Korle Bu Teaching Hospital, Accra, Ghana; 10 Department of Neurosurgery, Hannover Medical School, Carl-Neuberg-Strasse 1, 30625 Hannover, Germany

**Keywords:** Whole-Exome sequencing, Neurogenetics, Clinical genomics, Neurological disorders, Neurosurgery

## Abstract

Whole-exome sequencing (WES), a ground-breaking technology, has emerged as a linchpin in neurology and neurosurgery, offering a comprehensive elucidation of the genetic landscape of various neurological disorders. This transformative methodology concentrates on the exonic portions of DNA, which constitute approximately 1% of the human genome, thus facilitating an expedited and efficient sequencing process. WES has been instrumental in advancing our understanding of neurodegenerative diseases, neuro-oncology, cerebrovascular disorders, and epilepsy by revealing rare variants and novel mutations and providing intricate insights into their genetic complexities. This has been achieved while maintaining a substantial diagnostic yield, thereby offering novel perspectives on the pathophysiology and personalized management of these conditions. The utilization of WES boasts several advantages over alternative genetic sequencing methodologies, including cost-effectiveness, reduced incidental findings, simplified analysis and interpretation process, and reduced computational demands. However, despite its benefits, there are challenges, such as the interpretation of variants of unknown significance, cost considerations, and limited accessibility in resource-constrained settings. Additionally, ethical, legal, and social concerns are raised, particularly in the context of incidental findings and patient consent. As we look to the future, the integration of WES with other omics-based approaches could help revolutionize the field of personalized medicine through its implications in predictive models and the development of targeted therapeutic strategies, marking a significant stride toward more effective and clinically oriented solutions.

**Graphical Abstract:**

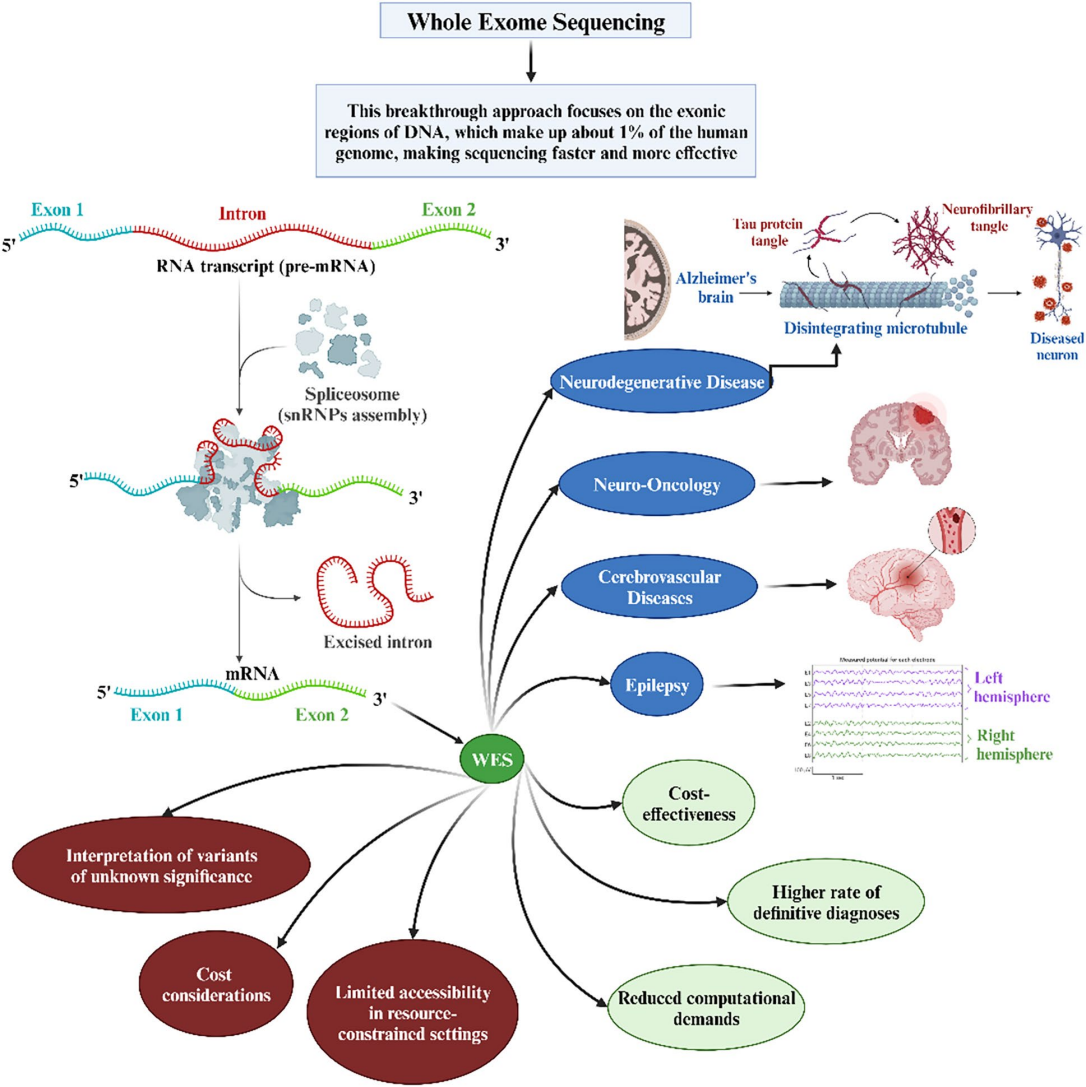

## Introduction

The emerging field of molecular diagnostics, utilizing deoxyribonucleic acid (DNA) and ribonucleic acid (RNA) molecules for disease diagnosis, has catalyzed advancements, such as whole-genome sequencing (WGS) and whole-exome sequencing (WES) [1, 2]. The intricate nature of neurological and neurosurgical conditions remains poorly understood, underscoring the imperative for a comprehensive genetic analysis [3]. While WGS offers a comprehensive analysis making it suitable for identifying both coding and non-coding pathogenic variations, and this increases the likelihood of incidental findings, including mutations not related to the patient’s present disease and variants with uncertain or incomplete effects [4]. Managing these incidental findings can be challenging and may require additional resources making it associated with higher costs as compared to WES [2, 4, 5]. WES addresses this limitation as it concentrates on coding exonic DNA, representing 1% of the genome and facilitating a more expedited sequencing process [6, 7]. As such, WES emerges as an indispensable, precise tool in clinical practice, addressing the complexities and heterogeneity inherent in these disorders and providing a tailored and efficient solution [3]. Specifically, WES is utilized to identify causative variants in monogenic disorders, genetic susceptibility factors, and somatic mutations [8–10]. Various mechanisms are employed in WES, with solid-based and liquid-based approaches being predominant in human exome sequencing [11, 12].

Exemplifying its efficacy, WES has notably discerned genetic variations associated with Mendelian diseases [6, 7]. The array-based method, as demonstrated by Hoischen et al. [13], has successfully identified cases of autosomal recessive ataxia. Additionally, Chou et al. [14] showcased the versatility of custom-based microarray WES in capturing both targeted and non-targeted parts of DNA in the study of neurofibromatosis. The strategic removal of these non-targeted DNA segments enhances the overall effectiveness of the method in delineating the genetic intricacies of neurofibromatosis [14]. Collectively, these instances underscore the demonstrated effectiveness and ongoing innovation of WES within the realm of medical molecular diagnostics.

This study aims to explore the multifaceted application of WES in the diagnosis, treatment, and research of neurological and neurosurgical conditions. By delving into the genetic intricacies of these disorders, we seek to contribute to the advancement of personalized medicine and pave the way for more effective clinical interventions. Through a thorough exploration of WES's role in neurology and neurosurgery, we aspire to provide valuable insights that contribute to the ongoing progress in molecular diagnostics and ultimately enhance patient care.

## Methodology

This review comprehensively assesses the application of WES in neurology and neurosurgery. The inclusion criteria for this review encompassed only full-text articles written in English. The time period is not specified to ensure the inclusion of all relevant articles. Both adult and pediatric populations are included. Several databases were employed to ensure an exhaustive literature search, including PubMed, EMBASE, Google Scholar, the Cochrane Library, and Scopus. Key terms, such as “whole-exome sequencing” and “next-generation sequencing”, were used in all searches, accompanied by additional terms comprising “Neurodegenerative Disorders”, “Cerebrovascular Disorders”, “Neuro-oncology”, “Brain tumours”, “Spinal cord tumours”, “Spinal cord diseases”, “strokes”, “epilepsy”, and “seizures”.

Additional sources were identified to augment the search strategy through a manual search of references cited in recent reviews focused on specific diseases. Rigorous exclusion criteria were adopted, involving the exclusion of standalone abstracts, case reports, posters, and unpublished or non-peer-reviewed studies. By instituting these criteria, the review sought to ensure the inclusion of high-quality and reliable evidence.

As for the scope of the review, no predetermined limit was set on the number of studies to be included, a strategy designed to gather comprehensive knowledge on the subject matter. The review included a range of study designs, including descriptive studies, animal-model studies, cohort studies, and observational studies. Moreover, it encompassed investigations conducted in pre-clinical and clinical settings, offering a broad perspective on the use of WES in neurology and neurosurgery. A summary of the methodology employed is presented in Table 1.

**Table 1:** Summary of methodology for this review

Methodology steps	Description
Literature search	PubMed, EMBASE, Google Scholar, the Cochrane Library, and Scopus
Inclusion criteria	- Full-text articles published in English - Focus on applications in neurology and neurosurgery - Adult and pediatric populations
Exclusion criteria	- Stand-alone abstracts - Case reports - Posters - Unpublished or non-peer-reviewed studies
Search Terms	Keywords, such as “whole exome sequencing” and “next-generation sequencing” coupled with indicators like “Neurodegenerative Disorders”, “Cerebrovascular Disorders”, “Neuro-oncology”, “Brain tumours”, “Spinal cord tumours”, “Spinal cord diseases”, “strokes”, “epilepsy”, and “seizures”
Additional search	- Manual examination of references cited in recent disease-specific reviews - No predetermined limit on the number of studies - Encompassing diverse study designs: • Descriptive studies • Animal model studies • Cohort studies • Observational studies - Including investigations in both pre-clinical and clinical settings

## History, evolution, and functional process

### History and evolution

In the 1970s, Frederick Sanger, a distinguished British chemist and Nobel laureate, introduced a transformative approach to DNA sequencing, known as Sanger sequencing [15]. This method relied on nucleotide-specific inhibitors to discern specific DNA sequences [15]. Over time, the technique underwent refinements and automation to enhance efficiency [16]. However, limitations persisted, including the sequencing of only one DNA fragment at a time and a maximum sequence length of 1000 base pairs. Notably, numerous experiments were required to identify candidate genes implicated in disease processes [16].

Between 1990 and 2003, the Human Genome Project successfully employed Sanger sequencing to sequence the entire human genome, involving the collaboration of 20 institutions and incurring a cost of approximately 3 billion dollars [16–18]. The primary goal was to identify common genetic regions linked to diseases, but the genetic heterogeneity observed in certain diseases rendered this approach less fruitful [16]. Diseases exhibited a spectrum of causative genes, some deviating from expected Mendelian inheritance patterns [16].

Recognizing the need for a more efficient and cost-effective sequencing method, researchers introduced next-generation sequencing (NGS) [19]. Unlike the all-encompassing approach of WGS, NGS offers versatility in its application. While NGS is often employed for selective sequencing, such as focusing on the exome—the coding region comprising approximately 1% of the total genome—this is just one of its many uses [7, 16]. NGS can be tailored to various sequencing goals, from sequencing the entire genome to targeting specific regions of interest. Moreover, NGS revealed that many Mendelian diseases could be attributed to genetic variations within the exome [20].

NGS revolutionized the field by providing a quicker, more cost-effective, and targeted method for identifying disease-causing genes. By 2011, the technology enabled the generation of a whole human genome sequence within weeks for 10,000 USD, overcoming the limitations associated with the complexity and genetic heterogeneity of certain diseases [16, 19, 21]. Within the realm of NGS, WES emerged as a pivotal advancement, representing a targeted and efficient method for decoding the protein-coding regions of the genome, ultimately enhancing our ability to understand and diagnose genetic disorders.

### Probe design and functional process

WES has become a vital tool in deciphering the genetic landscape of various diseases. This paper provides a comprehensive comparison of two widely employed WES methodologies: solution-based or liquid-phase sequencing and array-based or solid-phase sequencing [7, 12]. Here, we explore the procedural intricacies of each method, highlighting their advantages and limitations.

#### Solution-based exome sequencing

In the solution-based WES method, DNA samples undergo fragmentation, generating manageable fragments. Biotinylated probes, designed to hybridize with specific exonic regions, are introduced, facilitating target selection [7, 11]. Magnetic streptavidin beads aid in isolating the biotinylated probes, effectively washing away non-targeted genomic segments [12]. The incorporation of biotinylated probes and magnetic streptavidin beads allows for a highly selective targeting approach, ensuring efficient removal of non-targeted regions and minimizing both processing time and equipment requirements [7]. Consequently, the solution-based approach emerges as a time-efficient and resource-effective alternative in the realm of WES methodologies.

Amplification by polymerase chain reaction (PCR) enhances the quantity of the desired DNA segments for subsequent sequencing. The sequencing stage generates extensive genetic data, which is then subjected to bioinformatic analyses for interpretation [12]. This process can be divided into primary, secondary and tertiary sequencing. Primary analysis includes sequence generation and initial data quality control. This stage converts raw sequencing data into base calls. Additionally, it assesses the quality of sequencing reads and removes those of low quality to maintain high accuracy [22]. Secondary analysis entails bioinformatic procedures, such as aligning the raw sequence data before further examination. This includes aligning the filtered reads to a reference genome to identify genetic variants [22]. These variants are detected through computational methods, including those found in SAMtools and GATK [23]. These algorithms can detect a variety of variants including nucleotide substitutions, small insertions and deletions, and splicing variants. However, in order to detect copy number variations, tools such as Tools like CNVkit are normally used. Lastly, tertiary analysis involves interpretive tasks, such as annotating, filtering, prioritizing, and classifying variants, as well as case interpretation and reporting [22]. This stage assists in interpreting identified variants and predicting their clinical significance. Once these steps are completed, software tools like Picard can be used to mark duplicates [24, 25].

Even though sequencing technologies are advancing, there are still limitations to this technique. For example, gross rearrangements and triplet repeat expansions may be missed [26]. Therefore, while sequencing is a useful tool for genetic analysis, it is essential to take its limitations into consideration, and utilize other diagnostic techniques when necessary.

#### Array-based exome sequencing

Initially considered the primary method for WES, the array-based approach involves the use of high-density microarrays for probe binding [7, 27]. However, its adoption declined with the emergence of the solution-based method, which offered increased efficiency with reduced input data requirements [12]. Additionally, it demands extra time and equipment for array processing, potentially elongating the overall sequencing timeline [7].

As a result of these identified disadvantages, WES is commonly associated with solution-based capture methods rather than array-based capture. Prominent examples of platforms utilized for solution-based sequencing include Agilent SureSelect Human All Exon [24, 25, 28–31], NimbleGen SeqCap EZ Exome Library [32–35], Illumina TruSeq [36, 37], Illumina Rapid Capture Exome [37], and NimbleGen MedExome [37]. Each of these methodologies offers distinct advantages, including specificity and sensitivity, along with the capability to identify various variations, such as insertions and deletions [38–40].

WES technologies and platforms are crucial in advancing our comprehension of neurological and neurosurgical diseases. They contribute by elucidating the genetic underpinnings, uncovering new biomarkers, and elucidating relevant pathways. As WES technology progresses, it holds the potential to catalyze the creation of more accurate diagnostic tools and therapeutic approaches for intricate neurological conditions. The diversity of WES technologies employed in the study of neurological and neurosurgical diseases is outlined in Table 2 below.

**Table 2:** Summary of the types of WES technology in neurological and neurosurgical diseases

Platforms	Probe Design	Fragmentation Method	Advantages	Disadvantages
Agilent SureSelect Human All Exon [24, 25, 28–31, 38–41]	RNA	Ultrasonication	High throughput sequencing platform Excellent identification of insertions and deletions Fewer duplicate reads Greater alignment rate	Fewer high-quality reads Less uniform coverage
NimbleGen SeqCap EZ Exome Library [32–35, 38, 39, 41, 42]	DNA	Ultrasonication	Greatest bait density Efficient, with least sequencing needed to cover the target region Sensitive variant detection, with greater genotype sensitivity High specificity, with few off-target reads More uniform coverage in challenging exonic regions with high Guanine and Cytosine content	Not reported
Illumina TruSeq [36–38, 40]	DNA	Ultrasonication	Excellent detection of UTRs Excellent downstream identification of insertions and deletions	High percentage of off-target enrichment, reducing target efficiency Retains few reads after filtering
Illumina Rapid Capture Exome [37, 38, 40]	DNA	Transposomes	Excellent detection of UTRs	Coverage bias due to high Guanine and Cytosine content, leading to decreased overall uniformity Retains few reads after filtering
NimbleGen MedExome [37, 43]	DNA	Ultrasonication	Superior coverage in genes of high clinical relevance, enabling enhanced detection of relevant mutations associated with diseases Small drop-off rate	Less efficient, more sequencing required to cover target region

*DNA* Deoxyribonucleic acid, *RNA* ribonucleic acid, *UTR* untranslated region, *WES* whole-exome sequencing

## WES in neurological and neurosurgical disease management

### Neurodegenerative diseases

Significant advancements were made in understanding the genetic underpinnings of neurodegenerative diseases (NDs), specifically Alzheimer’s disease (AD), Parkinson’s disease (PD), amyotrophic lateral sclerosis (ALS), and spinocerebellar ataxia (SCA), facilitated by the application of WES. Through comprehensive analyses, WES has uncovered rare variants, novel mutations, and crucial insights into the genetic complexities associated with these debilitating conditions.

#### Alzheimer’s disease

WES has played a pivotal role in advancing our understanding of AD genetics, particularly in early-onset cases. Contrary to the expected prevalence of causative variants in major AD genes, such as *APP*, *PSEN1*, and *PSEN2*, WES has revealed a lower frequency in both familial late-onset AD and sporadic cases [25]. Moreover, WES studies have highlighted the significance of ultra-rare, loss-of-function variants in the *SORL1* gene, linking them to an earlier onset of AD [44]. Notably, analyses in both AD cases and controls have uncovered rare, damaging variants in genes associated with amyloid-β processing, lipid metabolism, and microglial function, providing insights into the multifaceted pathogenesis of AD [37]. Rare variants in known AD risk genes, such as *AKAP9*, *CD33*, and *CR1*, were also identified, pointing to links between AD, immunity, neuronal structure, and mitochondrial function. The discovery of genetic links across multiple families suggests potential significance in other neurological pathologies, such as Charcot–Marie–Tooth and other synapse dysfunctions [45].

It is important to distinguish between causative genes and risk factors in AD genetics. *APP, PSEN1*, and *PSEN2* are causative genes, meaning mutations in these genes directly lead to the development of AD. On the other hand, genes such as *SORL1*, as well as others mentioned above, are risk factors associated with the disease. These risk factors are identified through genome-wide association studies (GWAS), which help find genetic similarities associated with specific diseases, allowing for a deeper understanding of their genetic architecture.

#### Parkinson’s disease

In the realm of PD, WES has elucidated novel mutations in the *CSMD1* gene, a complement control protein associated with inflammation in the CNS and previously linked to PD risk [28]. These findings reinforce the potential of the complement pathway as a therapeutic target for PD. Among pure PD forms, *VPS35* and *VPS13C* are two genes discovered by WES [46, 47]. Many others are identified in complex forms of PD (*DNAJC6,* and *SYNJ1*), while others are awaiting confirmation (*CHCHD2*, *DNAJC13*) [46, 47]. Additionally, WES has identified enrichments in genes related to the extracellular matrix and regions previously implicated in PD by GWAS [48]. Notably, the gene *RAD51B*, known for its protein interaction with *RAD51*, has been associated with congenital mirror movements and comorbidities with PD [48].

GWAS have also provided deeper insights into PD’s genetic architecture. These studies analyze the entire genome to identify variations associated with the disease. Notable genetic risk factors include *SNCA, LRRK2*, and *MAPT*, which influence protein aggregation and mitochondrial function. For example, SNCA variants affect alpha-synuclein protein aggregation, a fundamental trait of PD, while more common mutations such as *LRRK2* affect kinase activity and neuronal health [49, 50].

Furthermore, additional genes such as *NRXN2* have been implicated in hereditary PD. A study involving a South African family with autosomal dominant PD identified a possible pathogenic mutation in the *NRXN2* gene using WES. The *NRXN2* variant showed consistency regarding absence within unaffected family members and controls, and was expressed in the substantia nigra [51]. The TMEM protein family genes (*TMEM230*, *TMEM59*, *TMEM108*) have also shown potential associations with PD, through their role in the regulation of vesicular trafficking and autophagy [52]. However, subsequent studies in other populations, including Chinese and Caucasian cohorts, have shown mixed results regarding the prevalence and significance of TMEM230 mutations [53].

Furthermore, the application of WES has expanded our understanding of early-onset PD genetics in specific ethnic populations, revealing new homozygous pathogenic variants in the *PRKN*, *PARK7*, and *PINK1* genes in Iranian patients [29]. Likewise, an investigation conducted among ethnic Chinese participants demonstrated that 7.5% exhibited pathogenic variants in established PD genes, yielding notable results in the identical genes observed in the Iranian cohort [54].

#### Other NDs

WES has been instrumental in delineating the genetic landscape of ALS and SCA. For ALS, at least 24 genes have been identified as associated with the disease. Among these, *KIF5, NEK1*, and *ATXN2* are notable risk factors. The identification of a *UBQLN2* mutation, unique to ALS without frontotemporal dementia, and its association with key neuronal proteins in inclusions underscore the specificity of WES in unveiling disease-specific mutations [36]. A noteworthy revelation establishes a connection between mutations in the valosin-containing protein (VCP) gene and ALS, extending its previously known associations with other NDs like body myopathy, Paget’s disease, and frontotemporal dementia [55]. In the case of SCA, WES has revealed a critical mutation in the *ITPR1* gene, linking it to both congenital non-progressive SCA and adult-onset SCA type 15 [56]. Additionally, WES has identified mutations in genes encoding voltage-gated potassium channels, highlighting the pivotal role of ion channels in regulating neuronal excitability and contributing to cerebellar degeneration [34].

Hereditary ataxias, including SCA, are a large group of neurodegenerative disorders which are defined by progressive cerebellar ataxia. These disorders are often caused by mutations in various genes responsible for maintaining cerebellar function and integrity. The identification of such mutations through WES has significantly advanced our understanding of the molecular mechanisms underlying these conditions.

Furthermore, WES has significantly improved diagnostic success. In a study of 76 diverse families with sporadic or familial cerebellar ataxia, excluding common SCAs and Friedreich ataxia, WES yielded definitive or probable diagnoses in 32% of the cases [57]. The most prevalent mutations that emerged were in the *RFC1*, *KIF1A*, and *SYNE1* genes. This underscores the efficacy of WES in uncovering the genetic underpinnings of relatively rare cerebellar ataxia within a diverse cohort [57]. Other hereditary ataxias include, Ataxia–Telangiectasia (A–T), caused by mutations in the *ATM* gene. This gene is responsible for DNA repair, meaning mutations often lead to progressive cerebellar degeneration, immunodeficiency, and an increased risk of cancer [58]. Although the *ATM* gene was identified prior to the advent of NGS, its large size made genetic diagnosis difficult; however, WES has improved the analysis of this gene, facilitating the molecular diagnosis of A–T. Finally, WES has advanced the analysis of causal genes for Wolfram syndrome (WFS). For example, recently, WES identified two novel homozygous variants in the *WFS1* gene in Moroccan families: a missense mutation (c.1329C>G; p.Ser443Arg) and a nonsense mutation (c.1113G>A; p.Trp371Ter) [59]. These variants, which affected conserved amino acid residues and were absent from genetic databases and Moroccan controls, were validated as pathogenic through bioinformatics analysis and molecular modeling. This application of WES not only pinpointed the specific genetic causes of WFS in these families but also expanded the known mutational spectrum of the disease, demonstrating WES’s efficacy in diagnosing and understanding rare genetic disorders [59].

**Fig. 1:**
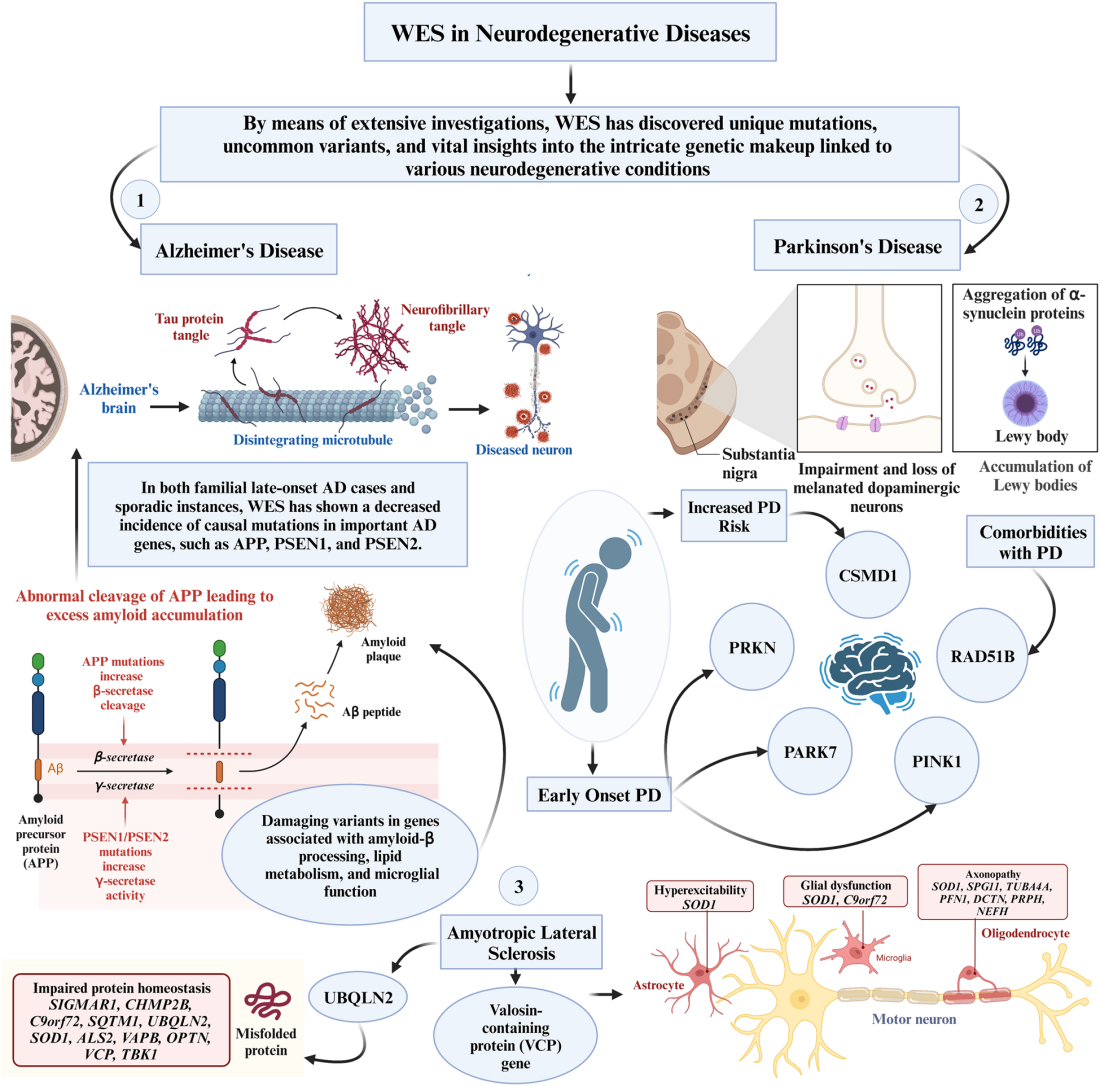
Role of whole-exome sequencing in neurodegenerative diseases. AD, Alzheimer’s Disease; ALS2, Alsin Rho Guanine Nucleotide Exchange Factor ALS2; APP, Amyloid Precursor Protein; C9orf72, Chromosome 9 Open Reading Frame 72; CHMP2B, Charged Multivesicular Body Protein 2B; CSMD1, CUB and Sushi Multiple Domains 1; DCTN, Dynactin; NEFH, Neurofilament, Heavy Polypeptide; OPTN, Optineurin; PARK7, Parkinsonism-Associated Deglycase (also known as DJ-1); PD, Parkinson’s Disease; PFN1, Profilin 1; PINK1, PTEN-Induced Putative Kinase 1; PRKN, Parkin RBR E3 Ubiquitin Protein Ligase (also known as PARK2); PRPH, Peripherin; PSEN1, Presenilin 1; PSEN2, Presenilin 2; RAD51B, RAD51 Paralog B; SIGMAR1, Sigma Non-Opioid Intracellular Receptor 1; SOD1, Superoxide Dismutase 1; SPG11, Spastic Paraplegia 11 (autosomal recessive); SQSTM1, Sequestosome 1; TBK1, TANK-Binding Kinase 1; TUBA4A, Tubulin Alpha 4a; UBQLN2, Ubiquilin 2; VAPB, Vesicle-Associated Membrane Protein, Associated Protein B and C; VCP, Valosin-Containing Protein; WES, Whole-Exome Sequencing

The collective findings stemming from WES applications in delineating the genetic landscapes of various NDs hold promise for therapeutic interventions. By uncovering rare variants, novel mutations, and disease-specific genetic signatures, WES offers avenues for developing targeted and personalized treatments for patients grappling with these complex conditions. The role of WES in NDs has been illustrated in Fig. 1.

### Cerebrovascular diseases

WES emerges as a powerful tool for deciphering the complex genetic underpinnings of cerebrovascular diseases (CVDs), offering unprecedented insights into diagnostic challenges and unveiling potential therapeutic targets [60].

#### Intracerebral aneurysms

WES has proven instrumental in identifying a spectrum of novel risk genes associated with intracranial aneurysms (IAs), elucidating key genetic contributors to CVDs. Notable instances include the identification of *EDIL3* and *TMEM132B* genes as potential risk genes through WES, with *EDIL3* substantiated by functional assessments and significant overexpression of the *TMEM132B* gene observed in IA tissue, linking both genes to the development and rupture of IAs [61, 62]. In a distinct ethnic context, the discovery of a missense variant (c.2519T>C, p.Leu840Pro) in the *NFX1* gene highlighted its specific association with a heightened prevalence of IAs in a Chinese family, underscoring the diverse genetic landscape of IA pathology [63]. Concurrently, potentially deleterious variants within the *PLOD3*, *NTM*, and *CHST14* genes were unveiled by WES, establishing them as primary causative factors for Familial IAs (FIAs) in Korean families [64]. Additionally, through GWAS and WES, more than 20 IA-candidate loci have been identified as risk factors. These collective findings underscore the indispensable role of WES in unraveling the intricate genetic landscape associated with IA.

#### Stroke

WES has played a pivotal role in advancing our comprehension of genetic factors linked to stroke, with a particular focus on ischemic stroke. Noteworthy genetic insights have been gleaned through the identification of mutations occurring in exon 11 of the *TRPV3* gene [35]. Additionally, two novel genes, *PDE4DIP* and *ACOT4*, have been associated with an elevated risk of ischemic stroke, providing novel dimensions to our understanding of the genetic basis of this CVD [65]. Furthermore, the investigation of a rare variation within the *PON* enzyme gene has revealed its capability to alter enzyme function, thereby increasing the susceptibility to ischemic stroke, particularly among individuals of African ancestry [66].

Sneddon syndrome, a rare genetic disorder, manifests as ischemic strokes predominantly affecting young individuals, particularly females [30]. It has elucidated that Sneddon syndrome is usually caused by bi-allelic *ADA2* gene pathogenic variants. Moreover, the impairment of *NOTCH3* signaling is not associated with Sneddon syndrome but is the causative gene of cerebral autosomal dominant arteriopathy with subcortical infarcts and leukoencephalopathy (CADASIL), a different genetic disorder characterized by recurrent strokes and dementia [30]. Moreover, the specificity of WES in dissecting genetic contributors to distinct CVD subtypes was underscored by the identification of noteworthy genetic variants. For instance, while deleterious variants in *KRIT1* and *NOTCH3* are well-known causes of intracerebral hemorrhage and strokes, respectively, the recognition of new *KRIT1* variants and rare *NOTCH3* variants, such as *p.R544C* for ischemic small vessel disease, highlights the precision of WES. This precision allows for the identification of previously unknown genetic variations associated with hemorrhagic and ischemic subtypes of CVDs [33]. These collective findings underscore the promising role of WES in identifying causative genes for specific subtypes of CVDs, offering prospects for personalized diagnostics and targeted therapeutic interventions.

#### Other CVDs

WES has been pivotal in advancing our understanding of a myriad of other CVDs. In a study focusing on subarachnoid hemorrhage (SAH), WES has pinpointed 30 SNPs in 17 genes, significantly enhancing our grasp of the genetic predispositions to SAH. Particularly, mutations in *TPO* and *PALD1* have been identified as novel risk factors, alongside an additional 25 genes that hint at key roles in extracellular matrix degradation and transcription factor signaling [31]. Similarly, WES has provided insights into cerebral small vessel disease (CSVD) and intracranial vertebral–basilar artery dissection (IVAD). For IVAD, an analysis of patients afflicted with isolated IVAD identified four known and seven novel variants in IVAD-related genes, as well as six variants in newly implicated genes [67]. In the context of CSVD, analysis of a Finnish patient cohort identified pathogenic variants in notable genes like *NOTCH3*, *HTRA1*, *COL4A1*, and *COL4A2* in a significant proportion of patients, also unearthing variants associated with other neurological disorders [68]. These same gene variants have also been recognized as causative genes of CVDs worldwide.

**Fig. 2:**
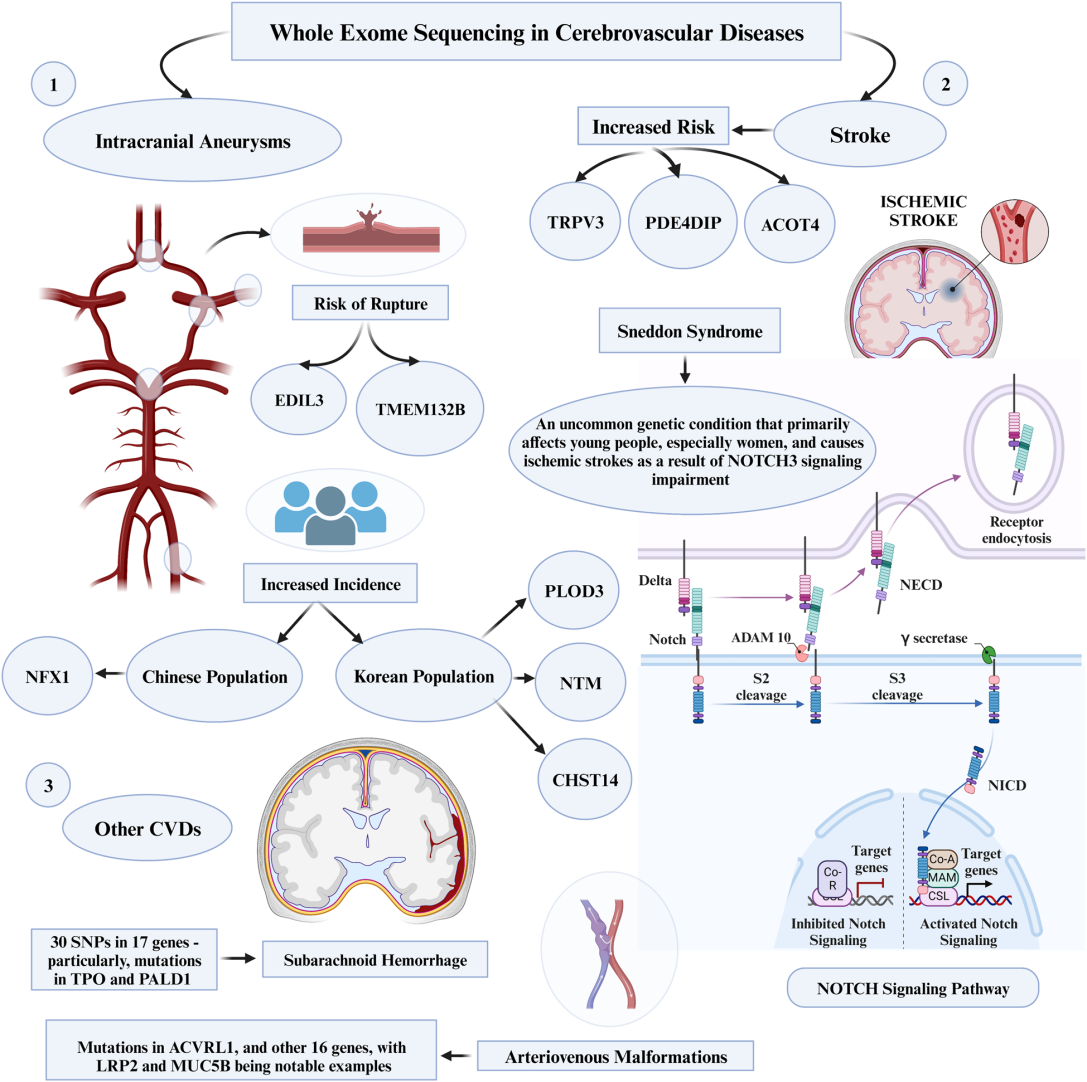
Role of whole-exome sequencing in cerebrovascular diseases. ACOT4, Acyl-CoA Thioesterase 4; CHST14, Carbohydrate Sulfotransferase 14; CVDs, Cardiovascular Diseases; EDIL3, EGF-Like Repeats and Discoidin I-Like Domains 3; LRP2, Low-Density Lipoprotein Receptor-Related Protein 2; MUC5B, Mucin 5B, Oligomeric Mucus/Gel-Forming; NECD, Notch Endocrine Complex Delta (Typically referred to as NOTCH1 or Notch Receptor 1); NFX1, Nuclear Transcription Factor, X-Box Binding 1; NICD, Notch Intracellular Domain (part of the Notch signaling pathway); NNTM, Nicotinamide Nucleotide Transhydrogenase (Typically referred to as NNT); PALD1, Phosphatase Domain Containing, Paladin 1; PDE4DIP, Phosphodiesterase 4D Interacting Protein; PLOD3, Procollagen-Lysine,2-Oxoglutarate 5-Dioxygenase 3; SNPs, Single-Nucleotide Polymorphisms; TMEM132B, Transmembrane Protein 132B; TPO, Thyroid Peroxidase; TRPV3, Transient Receptor Potential Cation Channel Subfamily V Member 3

In recent studies using WES to explore brain arteriovenous malformations (AVMs), significant genetic findings were uncovered. One study examined a Turkish family with three members having brain AVMs, and identified a *ACVRL1* mutation in two siblings, suggesting that WES is exceptionally useful in cases of locus heterogeneity [69]. Additionally, research in AVM cases focused on identifying rare genetic mutations led to the discovery of 16 genes with unique mutations, with *LRP2* and *MUC5B* being notable examples [70]. These findings highlight the potential of WES in understanding the genetics behind AVMs, offering new directions for research and treatment strategies in vascular diseases. The role of WES in CVDs has been illustrated in Fig. 2.

### Neuro-oncological diseases

In the realm of neuro-oncology, WES emerges as a transformative tool with significant implications for understanding and managing brain and spinal tumors. WES enables a comprehensive exploration of tumor genomics, addressing critical aspects such as tumor heterogeneity, the identification of diagnostic and therapeutic biomarkers, and the development of prognostic models. WES can be applied to two types of DNA: somatic and germline, each providing different insights into cancer genetics.

Somatic DNA refers to the genetic material found in the cells of the body, excluding the sperm and egg cells. Mutations in somatic DNA occur in specific cells during an individual's lifetime and are not inherited. These mutations can accumulate due to environmental factors, such as radiation and chemical compounds [71]. WES analysis of somatic DNA in tumors is essential for identifying mutations specific to cancer cells. This information helps researchers and clinicians understand the cancer's behavior and develop targeted therapies tailored to the genetic profile of the tumor [10].

In contrast, germline DNA is found in the sperm and egg cells and is inherited from one’s parents. Mutations in germline DNA are present in every cell of the body and can be transmitted to future generations. Germline mutations can predispose individuals to certain cancers, especially if the mutations include genes related to cancer development [72]. WES analysis of germline DNA is valuable for identifying inherited mutations that increase the risk of cancer. This information is essential for both assessing an individual's genetic predisposition to cancer, as well as for informing family members about potential risks [73].

#### Tumor heterogeneity and biomarkers

WES offers unparalleled insights into the genomic landscape of brain and spinal tumors, unraveling the intricacies of tumor heterogeneity. Glioblastoma multiforme (GBM) is the most frequent and lethal primary brain tumor [74]. WES aided in the genetic and molecular profiling of patient-derived xenograft (PDX), leading to the discovery that PDXs recapitulate many key phenotypic and molecular features of patient tumors. The PDX models capture most molecular drivers, including *TERT, EGFR, PTEN, TP53, BRAF*, and *IDH1*, and preserve most genetic driver alterations, including *EGFR* amplification, found in patient tumors [74]. In a rare variation of GBM named giant cell GBM (gcGBM), WES revealed recurrent mutations of *ATRX, PIK3R1, RB1* and *SETD2* [75]. The application of WES in diffuse glioma (DG) also revealed *TP53* and *ATRX* mutations, loss of function in *PTEN* and *EGFR* amplification, alongside *CDKN2A/B* deletion [24]. In pediatric patients with central nervous system (CNS) neoplasms, such as ependymoma, medulloblastoma and infiltrating astrocytoma, WES detected clinically pertinent Tier 1 (*BRAF* V600E, *NTRK* alterations, and *C19MC* amplification) and Tier 2 variants (*BRAF* fusion transcripts) [76]. The term “tier” refers to the classification of genetic variants based on their clinical relevance and potential impact on diagnosis, prognosis, and treatment. Tier 1 variants are those with strong clinical relevance, meaning they have significant implications for patient prognosis [76]. Tier 2 variants are defined as having moderate clinical relevance; they may have some evidence suggesting a potential impact on clinical outcomes, but this evidence is not conclusive. Tier 3 variants consist of variants of unknown significance that do not meet the criteria for Tiers 1 or 2 [76].

The application of WES in the study of brain metastases (BM) has yielded significant insights. Comparative analyses between BMs and primary tumors have uncovered noteworthy observations. Specifically, BMs exhibit a higher tumor mutational burden, characterized by elevated mutational signatures associated with homologous recombinant deficiency (HRD) and mismatch repair deficiency (MMRD) [77, 78]. In the context of colorectal cancer, frequent BM-specific mutations have been identified, encompassing *DDR, SCN7A, SCN5A, SCN2A, IKZF1,* and *PDZRN4* [77]. Conversely, in BM originating from triple-negative breast cancer (TNBC), TP53 mutations are prevalent, constituting the most frequently mutated gene in this context [78]. Moreover, lung cancer BM have exhibited mutations in *KMT2C* and *AHNAK2* [79]*.* These findings offer the potential for distinguishing the origin of BM, be it from breast cancer, lung cancer, or colorectal cancer. Furthermore, the novel metastasis-related mutations identified through WES hold promise as biomarkers for diagnostic and targeted therapeutic interventions.

WES has contributed significantly to advancing our comprehension of the genetic profile of sporadic vestibular schwannoma (VS), a benign tumor characterized by associated morbidities and diminished quality of life [32]. The findings underscore the marked heterogeneity within the genetic landscape of VS. Despite this diversity, a predominant pattern emerges wherein the majority of samples exhibit mutations either in the *NF2* gene or in genes closely associated with *NF2* [32]. Notably, the study establishes that Gamma Knife radiosurgery (GKRS) does not correlate with an elevated incidence of mutations in the context of VS [32]. Unraveling the exomic landscape allows for a precise characterization of molecular signatures, guiding the identification of specific pathways implicated in tumorigenesis.

**Fig. 3:**
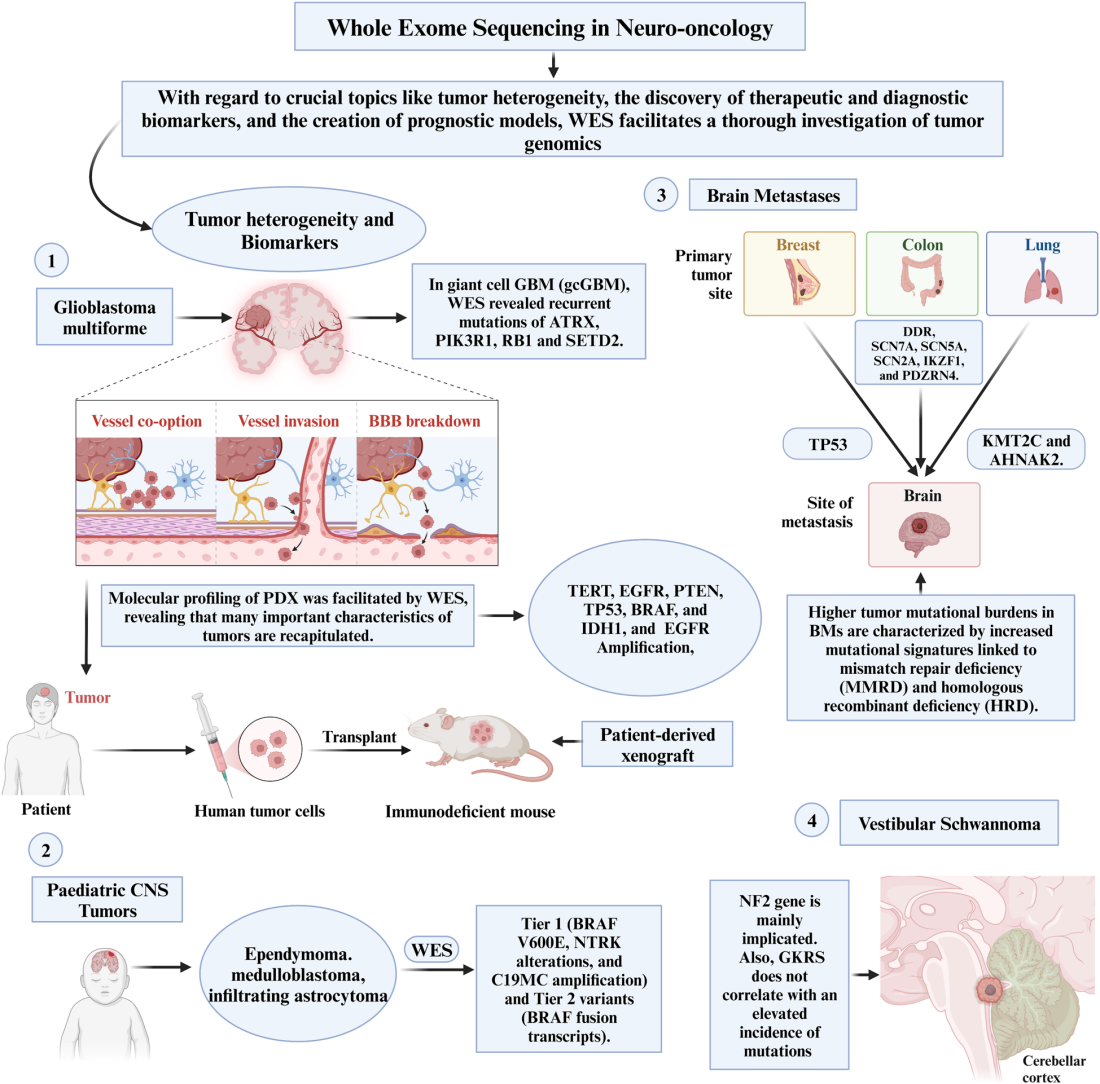
Role of whole-exome sequencing in neuro-oncological conditions. AHNAK2, AHNAK Nucleoprotein 2; ATRX, Alpha Thalassemia/Mental Retardation Syndrome X-Linked; BBB, Blood–Brain Barrier; BRAF, B-Raf Proto-Oncogene, Serine/Threonine Kinase; C19MC, Chromosome 19 MicroRNA Cluster; CNS, Central Nervous System; EGFR, Epidermal Growth Factor Receptor; gcGBM, Giant Cell Glioblastoma; GKRS, Gamma Knife Radiosurgery; IDH1, Isocitrate Dehydrogenase 1; KMT2C, Lysine Methyltransferase 2C; NTRK, Neurotrophic Receptor Tyrosine Kinase; PDX, Patient-Derived Xenograft; PIK3R1, Phosphoinositide-3-Kinase Regulatory Subunit 1; PTEN, Phosphatase and Tensin Homolog; RB1, Retinoblastoma 1; SCN2A, Sodium Voltage-Gated Channel Alpha Subunit 2; SCN5A, Sodium Voltage-Gated Channel Alpha Subunit 5; SCN7A, Sodium Voltage-Gated Channel Alpha Subunit 7; SETD2, SET Domain Containing 2; TERT, Telomerase Reverse Transcriptase; TP53, Tumor Protein p53; V600E, Valine replaced by Glutamic acid at position 600; WES, Whole-Exome Sequencing

These results collectively enhance our understanding of the genetic intricacies of brain tumors and provide valuable insights into the impact of interventions such as GKRS on its mutational landscape. By leveraging insights into tumor heterogeneity and biomarkers from WES, clinicians can tailor treatment strategies with heightened precision, facilitating the implementation of personalized therapeutic regimens. The role of WES in neuro-oncology has been illustrated in Fig. 3.

#### Prognostic models

The integration of WES-derived information into prognostic models refines patient stratification strategies, enabling a more personalized approach to treatment decisions. WES aided in the development of an immune-related prognostic signature (IPS) based on *PTEN*-associated-related genes [80]. Systematic analysis of the correlation of IPS with tumor immune cell infiltration and immune checkpoints revealed powerful prognosis prediction capacity in GBM with high sensitivity [80]. Furthermore, WES analysis of lung cancer BMs revealed a significant survival-associated mutation gene *ERF* [79]. WES data become integral in the development of prognostic models, contributing to more accurate predictions of disease outcomes.

In essence, the applications of WES in neuro-oncology not only deepen our understanding of the genomic intricacies underlying brain and spinal tumors but also pave the way for a new era of personalized and targeted therapeutic interventions.

### Spine diseases

The exploration of the interplay between genetic factors and spine diseases is a dynamic and evolving field of research [81]. Despite the progress, the molecular etiology of spinal diseases remains elusive due to the inherent genetic and phenotypic heterogeneity [82–85]. WES has emerged as a transformative tool, shedding light on the genetic landscape of spine disorders and significantly advancing our understanding of susceptibility, progression, and therapeutic avenues.

Spina bifida (SB), the second most prevalent nonlethal congenital malformation, has undergone unbiased WES analysis, revealing potential deleterious variants in candidate genes (partially identified on mouse models), such as *ATG2B*, *EWSR1*, *GPR83*, *IGBP1*, *MAML1*, *MTMR8*, *MAGI3*, *NUP205*, *PIK3R4*, *TSPEAR*, *TTC21A*, and *ZNF790* [86]. Congenital scoliosis (CS), characterized by lateral curvature and vertebral anomalies, poses a genetic challenge with a limited understanding of disease genes [83]. WES has identified de novo and homozygous variants (*SHISA3*, *AGBL5*, *HDAC4*, *PDE2A*, and *MOCOS*) contributing to CS development, alongside *LFNG* loss-of-function mutations implicated in a spectrum of spinal diseases [81, 83]. These novel gene findings offer potential insights for preventive and precision medicine, providing clinicians with a deeper understanding of the underlying pathology. However, the role of these specific genes related to CS, as identified in single families, requires further validation in order to confirm their role in CS.

Klippel–Feil syndrome (KFS), characterized by cervical vertebrae fusion, has revealed five novel genes through WES (*BAZ1B, FREM2, SUFU, VANGL1*, and *KMT2D*) with potential evidence for oligogenic inheritance [82]. SPONASTRIME dysplasia, a rare recessive skeletal disorder, has been elucidated through WES, uncovering bi-allelic mutations in the *TONSL* gene and providing critical insights into genome integrity and replication stress resistance [87]. WES has also unveiled potential causative genes for ossification of the posterior longitudinal ligament (OPLL), including *CYP4B1*, *NLRP1*, and *SSH2* [84]. *NLRP1* and *SSH2* are particularly significant due to their critical roles in inflammation, suggesting a potential link with the rapid growth of OPLL [84]. The utilization of WES has thus proven instrumental in advancing our comprehension of the predominantly unknown molecular etiology of spine diseases, addressing the challenges posed by genetic and phenotypic heterogeneity [82, 84].

In spondyloepimetaphyseal dysplasia (SEMD) with mental retardation (MR), WES has identified a variant (p.Asp237Gly) in the *AIFM1* gene [85]. Additionally, novel gene mutations associated with degenerative lumbar spinal stenosis (DLSS) have been uncovered, including *HLA-DRB1, PARK2, ACTR8, AOAH, BCORL1, MKRN2,* and *NRG4* [88]. Idiopathic scoliosis (IS), affecting healthy children, has demonstrated genetic associations through WES, revealing variants in *TNXB, CTNNA3, NTRK1*, and *PDE4DIP* [89]. The Kinesin family member 7 (KIF7)-dependent hedgehog signaling pathway has also been implicated in IS pathogenesis [90].

**Fig. 4:**
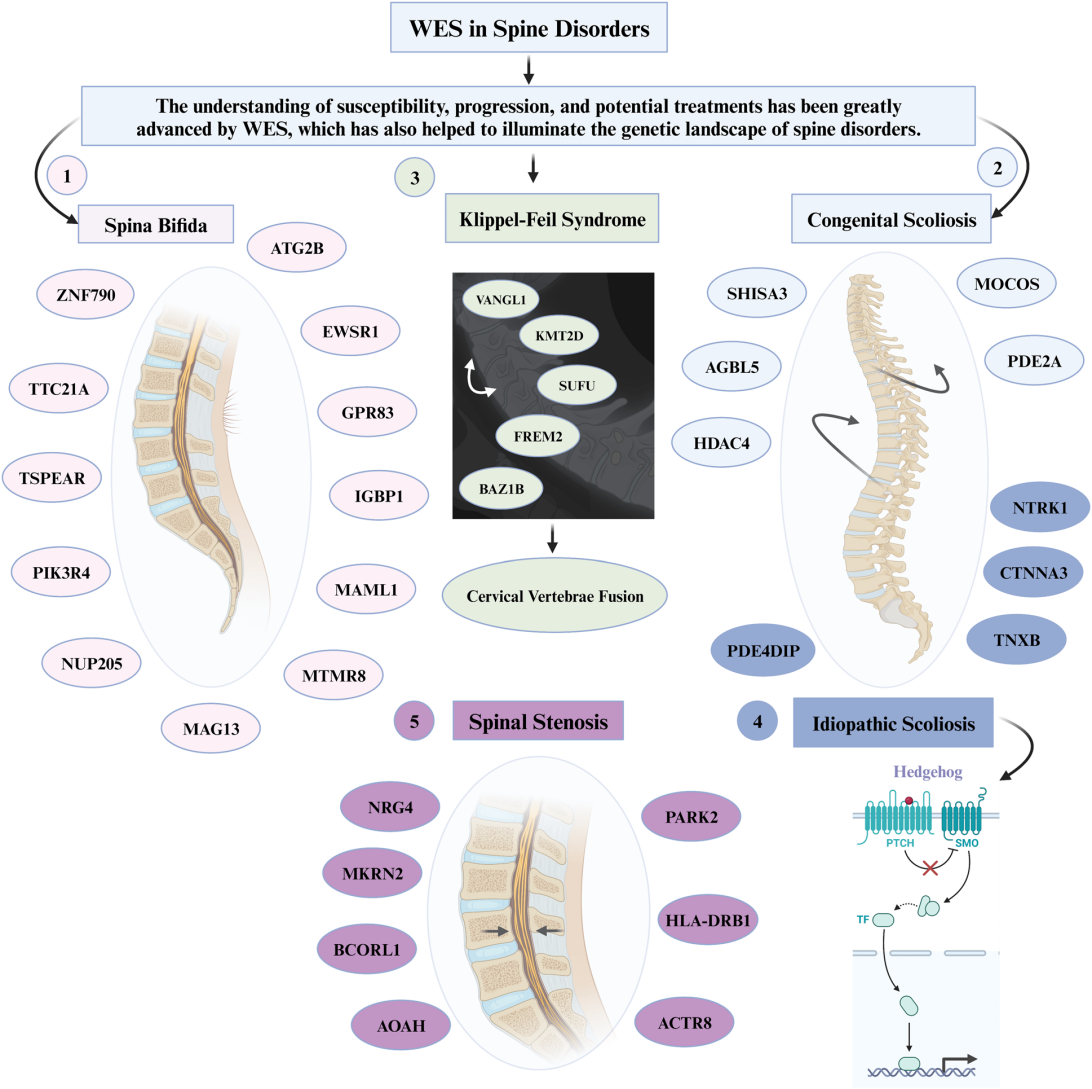
Role of whole-exome sequencing in spine diseases. ACTR8, ARP8 Actin-Related Protein 8 Homolog; AGBL5, ATP/GTP-Binding Protein Like 5; AOAH, Acyloxyacyl Hydrolase; ATG2B, Autophagy-Related 2B; BAZ1B, Bromodomain Adjacent To Zinc Finger Domain 1B; BCORL1, BCL6 Corepressor-Like 1; CTNNA3, Catenin Alpha 3; EWSR1, EWS RNA-Binding Protein 1; FREM2, FRAS1-Related Extracellular Matrix Protein 2; GPR83, G Protein-Coupled Receptor 83; HDAC4, Histone Deacetylase 4; HLA-DRB1, Human Leukocyte Antigen DR Beta 1; IGBP1, Immunoglobulin Binding Protein 1; KMT2D, Lysine Methyltransferase 2D (also known as MLL4); MAG13, Myelin-Associated Glycoprotein 13; MAML1, Mastermind-Like Transcriptional Coactivator 1; MKRN2, Makorin Ring Finger Protein 2; MOCOS, Molybdenum Cofactor Sulfurase; MTMR8, Myotubularin-Related Protein 8; NRG4, Neuregulin 4; NTRK1, Neurotrophic Receptor Tyrosine Kinase 1; NUP205, Nucleoporin 205; PARK2, Parkin RBR E3 Ubiquitin Protein Ligase; PDE2A, Phosphodiesterase 2A; PDE4DIP, Phosphodiesterase 4D Interacting Protein; PIK3R4, Phosphoinositide-3-Kinase Regulatory Subunit 4; PTCH1, Protein Patched Homolog 1; SHISA3, Shisa Family Member 3; SMO, Smoothened, Frizzled Class Receptor; SUFU, SUFU Negative Regulator of Hedgehog Signaling; TF, Transferrin; TNXB, Tenascin XB; TSPEAR, Thrombospondin-Type Laminin G Domain and EAR Repeats; TTC21A, Tetratricopeptide Repeat Domain 21A; VANGL1, VANGL Planar Cell Polarity Protein 1; ZNF790, Zinc Finger Protein 790

WES serves as an invaluable tool for uncovering novel biomarkers crucial for precise and targeted therapeutic interventions, facilitating personalized medicine in spine diseases. Furthermore, WES significantly contributes to scientific research endeavors, advancing our understanding of poorly understood spine diseases. It stands as a cornerstone in unraveling the genetic complexities of spine diseases, offering diagnostic precision, and paving the way for personalized medicine in spine surgery. The role of WES in spine diseases has been illustrated in Fig. 4.

### Epilepsy and seizure disorders

Epilepsy and seizure disorders, characterized by abnormal brain electrical activity [91], are increasingly elucidated through the lens of genetics, facilitated by WES. The utilization of this technology has revealed a diagnostic yield of 32.6% in patients with these disorders, uncovering fifteen mutations across fourteen genes, including both previously reported and novel variants in genes, such as *PAFAH1B1*, *LGI1*, and *SCN1A* [92]. In another instance, WES demonstrated even higher diagnostic yields: 40% for prospective cases and 30% for retrospective ones [93]. Furthermore, in patients whose molecular diagnoses were not explained through clinical genetic testing, pathogenic or likely pathogenic variants were identified in 25% through WES [94]. These underscore WES’s value in the clinical diagnosis of genetic diseases. Accurate diagnoses facilitated by WES significantly impact treatment decisions and prognostic assessments, enabling clinicians to predict disease progression, complications, and long-term outcomes [92]. Additionally, it enhances genetic counseling for patients and their families [95].

WES has unveiled a broad range of mutations and genetic variations linked to different types of epilepsy, surpassing the capabilities of traditional diagnostic methods [96]. This technique not only deepens our understanding of epilepsy's molecular mechanisms but also holds profound implications for personalized medicine. WES identified six novel mutations in the exon of the *SCN1A* gene (c.4224G>C, c.3744_3752del, c.209del, c.5727_5734delTTTAAAACinsCTTAAAAAG, c.5776delT, and c.1603C>T, p.Arg535Cys), in patients with epilepsy [97, 98]. In individuals affected by focal epilepsy, constituting 60% of all epilepsy cases, WES revealed multiple novel mutations within the *NPRL3* gene [99, 100]. This gene encodes a protein crucial for suppressing mammalian Target of Rapamycin (mTOR) signaling [99, 100]. The predominant genetic alteration identified in the *NPRL3* gene is characterized by a loss-of-function mutation [100].

In the pediatric domain, WES has played a pivotal role in unraveling critical biomarkers and fostering advancements in preventive and precision medicine. Within the cohort of patients experiencing developmental and epileptic encephalopathy (DEE), WES has revealed genetic variants in novel genes (*FGF12, GABBR1, GABBR2, ITPA, KAT6A, PTPN23, RHOBTB2, SATB2*) and candidate epilepsy-associated genes (*CAMTA1, FAT3, GABRA6, HUWE1, PTCHD1*) [94]. For infants and children with epilepsy, a spectrum of phenotypes was primarily attributed to *MMUT, KMT2A, MECP2, HIVEP2, TSCE, KCNQ2, POLG2, SYNGAP1, DGUOK, GALC, ARX, ADNP, COL3A1,* and *SCN2A* [101]. Novel variants in *SLC2A1, ANKRD11, GABRB3*, and *PACS1* were newly identified, enriching our understanding of the genetic landscape [102]. In the subset of children with post-neonatal epilepsy, WES brought forth candidate genes (*CACNA1H, CASK, RBFOX3*, and *RYR3*) potentially influencing susceptibility to epilepsy [8]. Moreover, it identified candidate genes (*KCNT1, MAGI2*, and *PRRT2*) linked to epilepsy syndromes characterized by incomplete penetrance and variable expressivity [8]. WES also pinpointed pathogenic truncating variants in the *IRF2BPL* gene among patients grappling with progressive myoclonus epilepsies (PMEs) typically presenting in late childhood [103]. These revelations underscore the substantial contribution of *IRF2BPL* to PME, urging its inclusion in genetic testing when PME is suspected. Additionally, WES spotlighted inherited pathogenic variants of *BRAT1*, resulting in a truncated BRAT1 and contributing to the development of lethal neonatal rigidity and multifocal seizure syndrome (RMFSL), a rare autosomal recessive neurological disease [104]. These insights not only advance clinical diagnoses but also provide essential genetic counseling and perinatal interventions for affected families.

In the realm of pathology, WES has proven invaluable, particularly in post-mortem examinations of brain tissue from individuals with Rasmussen encephalitis (RE), a neurological disorder intricately linked to epilepsy. A study by Leitner DF et al. (2023) showcased the application of WES in this context, revealing a spectrum of rare variants in genes, including *SCN1A, FCGR3B, MTOR, HLA-DRB1,* and *HLA-DQA2,* which were of unknown significance [105]. These findings provided insights into activated immune signaling pathways and immune cell type annotation enrichment, suggesting the involvement of innate and adaptive immune responses. Notably, identified human leukocyte antigen (HLA) variants may contribute to increased vulnerability to RE.

**Fig. 5:**
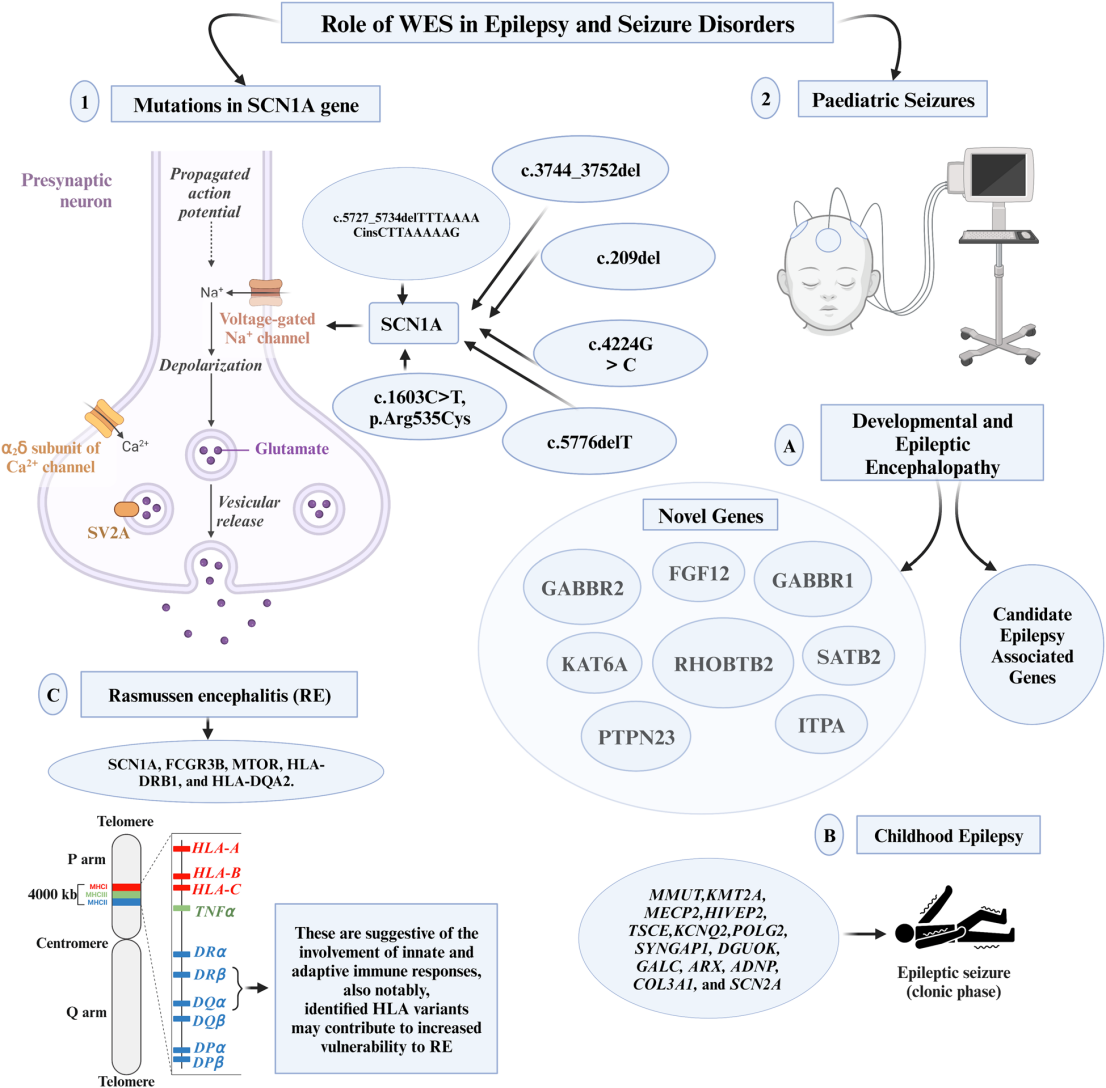
Role of Whole-Exome Sequencing in Epilepsy and Seizure Disorders. ADNP, Activity-Dependent Neuroprotector Homeobox; ARX, Aristaless-Related Homeobox; COL3A1, Collagen Type III Alpha 1 Chain; DGUOK, Deoxyguanosine Kinase; FCGR3B, Fc Fragment of IgG Receptor IIIb; FGF12, Fibroblast Growth Factor 12; GABBR1, Gamma-Aminobutyric Acid Type B Receptor Subunit 1; GABBR2, Gamma-Aminobutyric Acid Type B Receptor Subunit 2; GALC, Galactosylceramidase; HIVEP2, Human Immunodeficiency Virus Type I Enhancer-Binding Protein 2; HLA-DQA2, Human Leukocyte Antigen DQ Alpha 2; HLA-DRB1, Human Leukocyte Antigen DR Beta 1; KAT6A, Lysine Acetyltransferase 6A; KCNQ2, Potassium Voltage-Gated Channel Subfamily Q Member 2; KMT2A, Lysine Methyltransferase 2A; MECP2, Methyl CpG-Binding Protein 2; MMUT, Methylmalonyl-CoA Mutase; MTOR, Mechanistic Target of Rapamycin Kinase; POLG2, Polymerase (DNA) Gamma 2, Accessory Subunit; PTPN23, Protein Tyrosine Phosphatase, Non-Receptor Type 23; RHOBTB2, Rho-Related BTB Domain Containing 2; SATB2, SATB Homeobox 2; SCN1A, Sodium Voltage-Gated Channel Alpha Subunit 1; SCN2A, Sodium Voltage-Gated Channel Alpha Subunit 2; SV2A, Synaptic Vesicle Glycoprotein 2A; SYNGAP1, Synaptic Ras GTPase Activating Protein 1

The integration of WES, coupled with targeted variant prioritization, emerged as a pivotal approach in unveiling mutations in previously undiagnosed epilepsy patients. This underscores the potential of WES to uncover genetic factors that traditional diagnostic methods might overlook. The study highlights WES as a powerful tool not only in deciphering the genetic complexity of RE but also in shedding light on the intricate interplay between genetic variations and the immune system in the context of this neurological disorder. The role of WES in epilepsy and seizure disorders has been illustrated in Fig. 5.

## Advantages of WES over other genetic testing and sequencing methods

The rationale behind employing WES lies in the understanding that the protein-coding regions, constituting approximately 1% of the genome, harbor around 85% of genetic variants that significantly influence disease-related traits [106]. Consequently, WES presents a cost-effective and computationally efficient method to comprehensively analyze protein-altering genetic variations, surpassing alternative genetic testing methods such as WGS in these aspects [106].

As the cost of genetic testing decreases, WES is gaining prominence in clinical diagnostics, emerging as a primary alternative to gene panel testing for suspected genetic disorders. Preliminary analyses indicated that utilizing WES as an initial diagnostic tool could potentially reduce overall costs, particularly by obviating the need for supplementary tests [5]. Complementing this study, another comparative analysis revealed WES to be more cost-effective and diagnostically superior to gene panel testing for complex monogenic conditions in children [9]. WES proved to be more economical than the least costly gene panel in 26% of cases, while maintaining diagnostic success and missing only a maximum of 8% of clinically significant variants, establishing it as a superior and financially viable option for such diagnoses [9].

Moreover, the application of WES to intellectually disabled patients showcased both effectiveness and cost-efficiency. Identifying relevant diagnostic outcomes in 29.4% of cases, WES proved significantly more economical than traditional genetic diagnostic methods, underscoring its viability as a cost-effective diagnostic avenue [107]. Ewans et al. with a comparable cohort reported similar results, emphasizing that reanalysis of WES data after 12 months led to an increased diagnostic success rate [108]. This improvement was attributed to the inclusion of newly published disease genes, more comprehensive phenotype information, and enhanced bioinformatics. Additionally, the re-evaluation demonstrated cost savings for each additional diagnosis in the intellectual disability cohort [108].

Furthermore, WES stands out as an unbiased and valuable approach in the genetic diagnostics of neurological diseases, particularly in the context of neuromuscular disorders (NMDs) characterized by their diverse clinical and genetic manifestations [109]. Within a patient cohort exhibiting variations in ages of onset, neuromuscular phenotypes, and inheritance patterns, WES was employed as a follow-up strategy subsequent to inconclusive results from targeted gene testing. Notably, disease-causing variants were successfully identified in 19% of patients, with *COL6*-genes and *RYR1* emerging as principal contributors to the identified NMDs. Crucially, the unbiased nature of panel-based WES uncovered likely pathogenic variants in an additional 24% of cases [109]. This unbiased methodology of WES, implemented through NMD gene filtration, demonstrates its clinical efficacy as a primary diagnostic tool for NMDs. It provides holistic insights for precise molecular genetic diagnoses, facilitating subsequent clinical management, genetic counseling, and potential therapeutic interventions.

WGS offers comprehensive coverage of both coding and non-coding regions, improving diagnostic yield for Mendelian disorders by detecting structural variants, copy number variations, and novel gene-disease associations that WES might miss [4, 110]. WGS, however, is more expensive and generates vast amounts of data, requiring significant computational resources for storage and analysis. This leads to higher operational costs and longer turnaround times [4, 110]. Additionally, WGS increases the likelihood of incidental findings, complicating clinical decision-making, and interpreting variations in non-coding regions remains challenging [4, 110]. WES adopts a targeted approach by focusing on the coding regions of the genome, which are most likely to contain disease-causing mutations, resulting in a smaller data volume that simplifies analysis and interpretation. This targeted sequencing reduces the likelihood of incidental findings unrelated to the patient’s current condition, which is beneficial in clinical diagnostics [4]. Additionally, WES requires fewer computational resources for data storage and processing, leading to quicker turnaround times for data interpretation and clinical decision-making [4]. In the extant literature, the choice between WES and WGS depends on the specific clinical scenario, the need for comprehensive genomic coverage, cost considerations, and the available resources. WES is typically chosen for its cost-effectiveness and efficiency in coding regions, while WGS is preferred for its comprehensive coverage and higher diagnostic yield, particularly in complex or undiagnosed cases [4, 5, 111]. The advantages of WES over other genetic testing and sequencing methods are summarized in Table 3.

**Table 3:** Summary of the advantages of WES over other genetic testing and sequencing methods

Advantage	Description
Precision Targeting of Protein-Coding Regions [4, 106]	• WES targets and sequences coding regions of the genome, where disease-causing mutations are most likely to be found. This reduces data volume, simplifying analysis and interpretation • Generates less data than WGS, requiring fewer computational resources and leading to faster data processing and interpretation
Prominence in Clinical Diagnostics [5, 9, 108]	• Higher diagnostic rates in pediatric neurology compared to standard care • Diagnostically superior for complex monogenic conditions in children • Reanalysis after 12 months increases diagnostic success rates, attributed to updated genetic information and enhanced bioinformatics
Cost-effectiveness [5, 9, 107, 108]	• Cost-efficient in complex monogenic conditions in children and intellectually disabled patients • More economical than the least costly gene panel in 26% of cases, maintaining diagnostic success • Potential overall cost reduction using WES as an initial diagnostic tool, potentially eliminating the need for supplementary tests
Unbiased Diagnostic Approach [109]	• Unbiased approach in the genetic diagnostics of neurological diseases, particularly NMDs with diverse clinical and genetic manifestations • Employed as a follow-up strategy in patients with inconclusive results from targeted gene testing, WES successfully identified disease-causing variants, such as *COL6-*genes and *RYR1*, in 19% of patients • Unbiased methodology uncovers likely pathogenic variants in an additional 24% of cases, demonstrating clinical efficacy as a primary diagnostic tool

*WES* Whole-exome sequencing, *WGS* whole genome sequencing, *NMDs* neuromuscular disorders

## Challenges of WES in neurological applications

The implementation of WES in neurological applications brings forth multifaceted challenges, encompassing communication of results to patients and integration into electronic health records. These challenges coalesce primarily into three overarching categories: interpretation of variants, incidental findings, and cost-effectiveness.

### Challenges in identifying unknown variants

ACMG guidelines for interpreting sequence variants face challenges in assigning pathogenicity to novel variants, often resulting in classifications as “variants of uncertain significance” (VUS) [112]. Establishing genotype–phenotype correlations is complex, especially for genes not previously linked to the patient’s phenotype [112]. High levels of evidence, including functional studies and familial cosegregation, are needed to classify variants as pathogenic, particularly in asymptomatic individuals [112]. These challenges are not unique to WES but are common across other genomic techniques. In WES, while single-nucleotide alterations are relatively straightforward, complex insertions and deletions pose challenges, exacerbated by the diversity and in identification methods, leading to inconsistent findings [62]. The extensive generation of variants with unknown significance through WES poses a considerable challenge, complicating the interpretation process and presenting a bottleneck in clinical applications [113]. This complexity is exemplified in the context of drug-resistant epilepsy research, where the application of WES identified variables with unknown origins, thereby introducing complications into the study results [114].

The abundance of variants with uncertain implications highlights a critical hurdle in fully harnessing the potential of WES in a clinical context. This necessitates further refinement and collaborative efforts across specialties to optimize bioinformatics tools for interpreting new WES data [115]. The resulting uncertainty necessitates physicians to exercise judgment, potentially leading to increased false positives/negatives and unwarranted patient stress.

### Cost and inaccessibility

Despite WES being acknowledged as more cost-effective in comparison to other genetic testing and sequencing methods [107], its overall cost remains a significant concern, particularly in the context of prevalent neurological disorders like epilepsy or axonopathies, which hampers its broad implementation [116]. The financial considerations associated with WES encompass both data storage and interpretation [116]. For WES to become fully integrated into the healthcare system as the standard of care, it is imperative to substantiate its cost-effectiveness and efficacy to insurance companies [117]. The elevated costs pose a notable barrier, especially in low-income nations, underscoring the necessity for technological advancements that can alleviate storage and processing expenses.

### Ethical, legal, and social concerns

WES introduces several ethical considerations that necessitate meticulous examination. While WES offers the advantage of providing a definitive diagnosis, it is not without its associated negative psychosocial impacts. In the case of parents with children diagnosed with rare genetic diseases, the adverse psychosocial effects of WES test results encompass a diminishment of hope for recovery, necessitating substantial effort to reconstruct this social support system [118].

Incidental findings during WES, unveiling potentially significant genetic variants unrelated to the primary purpose of the test, present a notable ethical dilemma [119]. The revelation of such findings may induce unwarranted anxiety among patients and may be prone to misinterpretation by both patients and clinicians, highlighting the delicate balance between scientific discovery and its implications for healthcare decision-making [119]. An additional ethical facet pertains to informed consent, where patients must grasp the breadth and implications of the findings, demanding transparency in the outcomes of sequencing methods [120]. Privacy and data security become paramount, given that WES generates sensitive genetic information with potential risks to patient confidentiality, necessitating meticulous safeguarding of identifiable characteristics [121, 122].

The frequency of incidental findings can vary between 1 and 9% of the total assessed data [123]. Incidental findings that are actionable introduce ethical concerns, as untreated screened conditions may lead to patient harm. Nevertheless, patients frequently choose to forego incidental finding reports to evade the stress associated with discovering a genetic condition not initially screened for [123]. This raises a pertinent question: should physicians withhold information, foreseeing potential harm, to uphold patient autonomy?

The challenges of WES in neurological applications are summarized in Table 4.

**Table 4:** Summary of the challenges of WES in neurological applications

Challenges	Description
Challenges in identifying unknown variants	
Absence of Standardized Guidelines [62]	• Lack of standardized guidelines, especially in deciphering complex insertions and deletions • Diversity in identification methods leads to inconsistent findings, complicating interpretation
Variants of Unknown Significance [113, 114]	• WES generates numerous variants with unknown significance, posing challenges in interpretation and impeding clinical applications • Complexity is evident in drug-resistant epilepsy research, where WES identified variables with unknown origins, introducing complications into study results
Uncertainty and Clinical Applications [115]	• Variants' uncertainty requires refining bioinformatics tools for interpreting new WES data • Physician judgment becomes crucial, potentially leading to increased false positives/negatives and unwarranted patient stress
Cost and inaccessibility	
Overall Cost Concerns [107, 116]	• Despite being considered more cost-effective compared to alternative methods, cost remains a concern, particularly in prevalent neurological disorders • Financial considerations encompass data storage and interpretation
Insurance and Accessibility [117]	• To become a healthcare standard, WES must substantiate its cost-effectiveness and efficacy to insurance companies • Elevated costs pose barriers, especially in low-income nations
Ethical, legal, and social concerns	
Psychosocial Impact [118]	• WES results in adverse psychosocial effects for parents with children diagnosed with rare genetic diseases
Incidental Findings and Ethical Dilemma [119, 123]	• Incidental findings unveil potentially significant genetic variants unrelated to the primary purpose • Physicians face a dilemma regarding disclosing incidental findings, balancing patient autonomy and potential harm
Informed Consent and Privacy [120–122]	• Informed consent is crucial for patients to grasp the breadth and implications of findings, demanding transparency • Privacy and data security are paramount, given that WES generates sensitive genetic information, necessitating meticulous safeguarding

*WES* whole-exome sequencing

## Discussion and future prospects

### Integration of WES with other omic approaches

An intriguing avenue of exploration involves linking genetic variants identified through WES with epigenetic alterations, unraveling their impact on DNA methylation, histone modifications, and chromatin structure [124]. This integration aims to associate identified genetic variants with gene expression patterns, providing insights into their influence on the cellular transcriptional landscape [125]. By merging genomic data with protein expression profiles, researchers can explore downstream effects on cellular processes and pathways, fostering a more comprehensive understanding of molecular mechanisms [125]. This holistic approach extends to connecting genomic variations with alterations in metabolic pathways, elucidating how genetic variants influence metabolite levels and the overall metabolic profile [126].

### Personalized medicine

The integration of multi-omics data plays a pivotal role in tailoring treatment plans to a personalized level, discerning patient-specific molecular profiles crucial for informed and targeted therapeutic interventions [127]. The future trajectory of WES is intricately linked to the evolution of personalized medicine. This trajectory envisions the synergy of WES and big data, presenting individual patients with terabytes of information, facilitating the derivation of correlations among diverse datasets to predict disease progression and precisely identify causative factors [128].

WES exhibits particular allure in clinical settings due to its coverage of actionable genomic regions, emphasizing variations in exon regions to pinpoint variants associated with disease-causing mutations. This has led to an exponential increase in WES data generation at the population level. WES seamlessly integrates into various sequencing projects, contributing to the classification of population variants and the identification of diseases linked to rare variants, ultimately augmenting the realm of personalized medicine [129]. This integration involves aligning the genetic makeup data of patients with tailored treatments, enhancing treatment efficacy. Noteworthy advancements in the intersection of WES and personalized medicine, particularly in neurology, are exemplified by the monoclonal antibody pembrolizumab [129]. This immune checkpoint inhibitor, currently under clinical trial for GBM, targets tumors expressing *PD-1*/*PD-L1*, evident in GBM [130].

However, the assimilation of WES into mainstream medicine for personalized treatment might encounter a timeline of approximately another decade. Clinicians exhibit hesitancy in embracing NGS findings into routine patient management, citing concerns related to complexity and the perceived lack of “clinician-friendly” data. This cautious approach underscores the necessity for continued efforts in simplifying and streamlining the integration of WES into clinical practice [128].

### Predictive models

Predictive models, leveraging integrated omics data and deploying machine learning algorithms, play a pivotal role in foreseeing disease risk, progression, and potential responses to specific treatments [131]. WES has emerged as a potent tool in this context, offering insights into genes associated with the onset of brain cancers and NDs [132].

Ongoing studies are actively engaged in predicting and altering the trajectories of devastating diseases through genetic confirmation. Notably, successful models have been developed for conditions, such as DOPA-responsive dystonia, glucose transporter type 1 deficiency, and X-linked adrenoleukodystrophy [133]. WES not only facilitates personalized treatment strategies for these diseases but also enables the implementation of preventative measures before serious effects manifest. An illustrative example is found in the screening of pediatric neurological diseases, specifically targeting epilepsy and neurometabolic disorders, aiming to preclude their onset in later years [133].

WES also demonstrates utility in predicting one of the most common NDs, PD. While the etiology of PD often involves a complex interplay between genetics and environmental factors, families with a monogenic inheritance pattern have been subjected to WES analysis. This approach aims to delineate the genes responsible for the hereditary forms of the disease [46]. Although PD remains incurable, physicians can assist families harboring these hereditary genes in implementing lifestyle changes that may mitigate or delay the onset of the disease [46]. Particularly intriguing is WES’s application in uncovering new genes implicated in various neurological disorders. This involves scrutinizing multiple generations within a family to pinpoint potential pathologically significant variants. Recent years have witnessed the identification and association of variants, such as *DNAJC13, CHCHD2, VPS35* (autosomal dominant), *VPS13C, DNAJC6,* and *SYNJ1* (autosomal recessive), with PD [46, 47]. GWAS have also provided deeper insights into PD’s genetic architecture. Identifying genetic risk factors, such as *SNCA, LRRK2*, and *MAPT*, influence protein aggregation and mitochondrial function [49, 50]. In essence, the accurate genome diagnosis facilitated by WES empowers physicians to identify latent phenotypes of rare neurogenetic diseases, enabling the provision of preventive treatments and contributing to proactive healthcare strategies.

### Addressing computational challenges

Addressing the computational challenges inherent in integrating diverse omics data stands as a pivotal concern, necessitating the development of robust computational frameworks to enable seamless integration. The effective management and analysis of large-scale, intricate datasets demand the implementation of scalable algorithms and the utilization of cloud computing resources [134]. Tackling the complexity of interpreting integrated omics data involves the ongoing development of visualization tools and statistical methods, strategically designed to enhance the interpretability of these intricate multidimensional datasets. To mitigate the lack of standardization in omics data formats, efforts are underway to promote and adopt standardized data formats and metadata, ensuring uniformity and comparability across various studies [134].

Moreover, the discovery of variants of uncertain significance occurs at a rate surpassing computational predictors. Consequently, clinicians grapple with the challenge of comprehending the significance of specific variants that may bear actionable consequences [135]. However, a potential solution lies in the application of machine learning methods, allowing the development of algorithms capable of predicting the clinical impact of rare variants on protein function [135]. Artificial intelligence (AI) holds promise in improving the statistical computation aspects of WES, provided it has access to a substantial amount of data. Achieving accurate genotype–phenotype connections through AI requires extensive datasets. Developers are already designing more effective causal algorithms to link sequence variants with biological phenotypes, enabling computers to precisely analyze omics data derived from WES [136]. This integration of advanced computational tools holds promise in enhancing the clinical utility of omics data, bridging the divide between genetic variation and actionable insights for improved patient care.

### Recommendations for further research

In the realm of multi-omics research, future investigations should delve into longitudinal studies, providing dynamic insights into the temporal dimensions of biological processes. The importance of validating findings through functional experiments cannot be overstated as it is crucial for comprehending the biological relevance of observed associations in multi-omics data [127]. Achieving a comprehensive understanding of the intricate relationship between molecular signatures and clinical outcomes necessitates the integration of multi-omics data with clinical information. To enhance the generalizability of research findings, there is an urgent call for studies to broaden their participant base, encompassing individuals from diverse ethnic backgrounds and geographical locations [127].

Furthermore, the ethical dimensions of multi-omics research should be a focal point, with a dedicated effort to address implications and establish robust privacy measures. Ensuring the responsible and ethical conduct of studies involving sensitive genetic and health information is paramount in safeguarding participant confidentiality and maintaining public trust in the scientific research process [127]. This holistic approach not only advances the scientific understanding of multi-omics but also underscores the commitment to ethical principles, reinforcing the integrity and credibility of research outcomes.

In the domain of pharmacogenomics, where the genetic makeup influences drug responses, an increasing number of pharmaceutical companies are leveraging WES. As genomics becomes more prevalent, and integration with imaging, medical health data, and mobile health data advances, the field is transitioning into the era of big data [137]. However, the integration of WES into big data presents challenges in terms of cost and security, necessitating large, well-protected data centers to store private information. Advancements in technology are pivotal for lowering the cost of WES and accelerating the turnaround time for routine sequencing [137]. The ongoing exploration of these avenues is essential for pushing the boundaries of knowledge and harnessing the full potential of genomics in healthcare. The discussions and prospects of WES in neurological applications are summarized in Table 5.

**Table 5:** Summary of future prospects of WES in neurological applications

Future prospects	Description
Integration of WES with other omic approaches	
Linking Genetic Variants with Epigenetic Alterations and Protein Expression Profiles [124–126]	• Explore the impact of genetic variants identified through WES on DNA methylation, histone modifications, and chromatin structure • Associate genetic variants with gene expression patterns, providing insights into their influence on downstream cellular processes and pathways
Personalized medicine	
Personalized Molecular Profiles [127, 128]	• Envision the synergy of WES, multi-omics data, and big data for deriving correlations and predicting disease progression, to tailor treatment plans to patient-specific molecular profiles for targeted therapeutic interventions
Translational Medicine [128, 129]	• WES covers actionable genomic regions, contributing to the classification of population variants and identification of diseases linked to rare variants • Notable advancements in WES and personalized medicine showcased by the approval of pembrolizumab in treating primary GBM • Continued efforts needed to simplify and streamline the integration of WES into clinical practice, to eliminate hesitancy of clinicians in integrating NGS findings into routine patient management
Predictive models	
Predicting Disease Risk and Progression [46, 132, 133]	• WES offers insights into genes associated with the onset of brain cancers and NDs • Successful models developed for conditions like DOPA-responsive dystonia, glucose transporter type 1 deficiency, and X-linked adrenoleukodystrophy • WES aids in predicting PD, offering insights into hereditary forms
Addressing computational challenges	
Developing Robust Computational Frameworks [134–136]	• Implement scalable algorithms and utilize cloud computing resources for effective management and analysis of large-scale datasets • Machine learning methods offer a potential solution for interpreting variants of uncertain significance, enhancing clinical utility • Development of more effective causal algorithms to link sequence variants with biological phenotypes, enabling precise analysis of omics data derived from WES
Visualization Tools and Statistical Methods [134]	• Ongoing development of visualization tools and statistical methods to enhance interpretability of integrated omics data
Standardization Efforts in Omics Data Formats [134]	• Promote and adopt standardized omic data formats to ensure uniformity and comparability across various studies
Recommendations for further research	
Longitudinal Studies and Functional Experiments [127]	• Prioritize longitudinal studies for dynamic insights into the temporal dimensions of biological processes • Validate findings through functional experiments to comprehensively understand the biological relevance in multi-omics data • Integrate multi-omics data with clinical information for a comprehensive understanding of the relationship between molecular signatures and clinical outcomes • Broaden the participant base, encompassing diverse ethnic backgrounds and geographical locations for enhanced generalizability
Ethical Dimensions and Privacy Measures [127]	• Dedicate efforts to address implications and establish robust privacy measures, especially in studies involving sensitive genetic and health information
Transition to the Era of Big Data in Genomics [137]	• Transition of the genomics field into the era of big data with integration with imaging, medical health data, and mobile health data advancements • Advancements in technology are pivotal for lowering the cost of WES associated with the security costs of big data and accelerating the turnaround time for routine sequencing

*DNA* Deoxyribonucleic acid, *GBM* glioblastoma multiforme, *ND* neurodegenerative disease, *NGS* next-generation sequencing, *PD* Parkinson’s disease, *WES* whole-exome sequencing

## Conclusion

WES represents a transformative advancement in the understanding and management of neurological and neurosurgical disorders, offering a profound synthesis of multi-omic insights. Its application ranges from unraveling the complexities of NDs to decoding the genetic underpinnings of cerebrovascular and neuro-oncological conditions, thereby guiding personalized medicine approaches. WES embodies the future of precision medicine, leveraging targeted genetic analysis to pave the way for tailored therapeutic strategies and predictive models, thereby enhancing patient care in the realm of neurology and neurosurgery.

## Data Availability

No datasets were generated or analysed during the current study.

## Abbreviations

AD: Alzheimer’s disease
AI: Artificial intelligence
ALS: Amyotrophic lateral sclerosis
A–T: Ataxia–Telangiectasia
AVM: Arteriovenous malformation
BM: Brain metastases
CADASIL: Cerebral autosomal dominant arteriopathy with subcortical infarcts and leukoencephalopathy
CNS: Central nervous system
CS: Congenital scoliosis
CSVD: Cerebral small vessel disease
CVD: Cerebrovascular disease
DEE: Developmental and epileptic encephalopathy
DG: Diffuse glioma
DLSS: Degenerative lumbar spinal stenosis
DNA: Deoxyribonucleic acid
DRPLA: Dentatorubral-Pallidoluysian Atrophy
FRDA: Friedreich’s Ataxia
FIA: Familial intracranial aneurysms
GBM: Glioblastoma multiforme
gcGBM: Giant cell glioblastoma multiforme
GKRS: Gamma Knife radiosurgery
GWAS: Genome-wide association studies
HLA: Human leukocyte antigen
HRD: Homologous recombinant deficiency
IA: Intracranial aneurysms
IPS: Immune-related prognostic signature
IS: Idiopathic scoliosis
IVAD: Intracranial vertebral–basilar artery dissection
KFS: Klippel–Feil syndrome
KIF7: Kinesin family member 7
MMRD: Mismatch repair deficiency
MR: Mental retardation
mTOR: Mammalian Target of Rapamycin
ND: Neurodegenerative diseases
NGS: Next-generation sequencing
NMD: Neuromuscular disorder
OPLL: Ossification of the posterior longitudinal ligament
PCR: Polymerase chain reaction
PD: Parkinson’s disease
PDX: Patient-derived xenograft
PME: Progressive myoclonus epilepsy
RE: Rasmussen encephalitis
RMFSL: Lethal neonatal rigidity and multifocal seizure syndrome
RNA: Ribonucleic acid
SAH: Subarachnoid hemorrhage
SB: Spina bifida
SCA: Spinocerebellar ataxia
SEMD: Spondyloepimetaphyseal dysplasia
TNBC: Triple-negative breast cancer
UTR: Untranslated region
VCP: Valosin-containing protein
VS: Vestibular schwannoma
VUS: Variants of uncertain significance
WES: Whole-exome sequencing
WGS: Whole-genome sequencing

## References

[1] Lee H, Tang H. Next-generation sequencing technologies and fragment assembly algorithms. Methods Mol Biol. 2012;855:155–74. 10.1007/978-1-61779-582-4_5. 22407708 10.1007/978-1-61779-582-4_5

[2] Udine E, Jain A, van Blitterswijk M. Advances in sequencing technologies for amyotrophic lateral sclerosis research. Mol Neurodegener. 2023;18(1):4. 10.1186/s13024-022-00593-1. (**Published 2023 Jan 13**). 36635726 10.1186/s13024-022-00593-1 PMC9838075

[3] Jiang T, Tan MS, Tan L, Yu JT. Application of next-generation sequencing technologies in neurology. Ann Transl Med. 2014;2(12):125. 10.3978/j.issn.2305-5839.2014.11.11. 25568878 10.3978/j.issn.2305-5839.2014.11.11 PMC4260045

[4] Meienberg J, et al. “Clinical sequencing: is WGS the better WES? Hum Genet. 2016;135(3):359–62. 10.1007/s00439-015-1631-9. 26742503 10.1007/s00439-015-1631-9 PMC4757617

[5] Vissers LE, Van Nimwegen KJ, et al. A clinical utility study of exome sequencing versus conventional genetic testing in pediatric neurology. Genet Med. 2017;19(9):1055–63. 10.1038/gim.2017.1. 28333917 10.1038/gim.2017.1 PMC5589982

[6] Seaby EG, Pengelly RJ, Ennis S. Exome sequencing explained: a practical guide to its clinical application. Brief Funct Genomics. 2016;15(5):374–84. 10.1093/bfgp/elv054. 26654982 10.1093/bfgp/elv054

[7] Teer JK, Mullikin JC. Exome sequencing: the sweet spot before whole genomes. Hum Mol Genet. 2010;19(R2):R145–51. 10.1093/hmg/ddq333. 20705737 10.1093/hmg/ddq333 PMC2953745

[8] Numis AL, da Gente G, Sherr EH, Glass HC. Whole-exome sequencing with targeted analysis and epilepsy after acute symptomatic neonatal seizures. Pediatr Res. 2022;91(4):896–902. 10.1038/s41390-021-01509-3. 33846556 10.1038/s41390-021-01509-3 PMC9064802

[9] Dillon OJ, Lunke S, Stark Z, et al. Exome sequencing has higher diagnostic yield compared to simulated disease-specific panels in children with suspected monogenic disorders. Eur J Hum Genet. 2018;26(5):644–51. 10.1038/s41431-018-0099-1. 29453417 10.1038/s41431-018-0099-1 PMC5945679

[10] Lawrence MS, Stojanov P, Polak P, Kryukov GV, Cibulskis K, Sivachenko A, Carter SL, Stewart C, Mermel CH, Roberts SA, Kiezun A, Hammerman PS, McKenna A, Drier Y, Zou L, Ramos AH, Pugh TJ, Stransky N, Helman E, Kim J, Sougnez C, Ambrogio L, Nickerson E, Shefler E, Cortés ML, Auclair D, Saksena G, Voet D, Noble M, Di Cara D, Lin P, Lichtenstein L, Heiman DI, Fennell T, Imielinski M, Hernandez B, Hodis E, Baca S, Dulak AM, Lohr J, Landau DA, Wu CJ, Melendez-Zajgla J, Hidalgo-Miranda A, Koren A, Mccarroll SA, Mora J, Crompton B, Onofrio R, Parkin M, Winckler W, Ardlie K, Gabriel SB, Roberts CWM, Biegel JA, Stegmaier K, Bass AJ, Garraway LA, Meyerson M, Golub TR, Gordenin DA, Sunyaev S, Lander ES, Getz G. Mutational heterogeneity in cancer and the search for new cancer-associated genes. Nature. 2013;499(7457):214–8. 10.1038/nature12213. (**Epub 2013 Jun 16**). 23770567 10.1038/nature12213 PMC3919509

[11] Gnirke A, Melnikov A, Maguire J, et al. Solution hybrid selection with ultra-long oligonucleotides for massively parallel targeted sequencing. Nat Biotechnol. 2009;27(2):182–9. 10.1038/nbt.1523. 19182786 10.1038/nbt.1523 PMC2663421

[12] Warr A, Robert C, Hume D, Archibald A, Deeb N, Watson M. Exome sequencing: current and future perspectives. G3 (Bethesda). 2015;5(8):1543–50. 10.1534/g3.115.018564. (**Published 2015 Jul 2**). 26139844 10.1534/g3.115.018564 PMC4528311

[13] Hoischen A, Gilissen C, Arts P, et al. Massively parallel sequencing of ataxia genes after array-based enrichment. Hum Mutat. 2010;31(4):494–9. 10.1002/humu.21221. 20151403 10.1002/humu.21221

[14] Chou LS, Liu CS, Boese B, Zhang X, Mao R. DNA sequence capture and enrichment by microarray followed by next-generation sequencing for targeted resequencing: neurofibromatosis type 1 gene as a model. Clin Chem. 2010;56(1):62–72. 10.1373/clinchem.2009.132639. 19910506 10.1373/clinchem.2009.132639

[15] Sanger F, Nicklen S, Coulson AR. DNA sequencing with chain-terminating inhibitors. Proc Natl Acad Sci U S A. 1977;74(12):5463–7. 10.1073/pnas.74.12.5463. 271968 10.1073/pnas.74.12.5463 PMC431765

[16] Petersen BS, Fredrich B, Hoeppner MP, Ellinghaus D, Franke A. Opportunities and challenges of whole-genome and -exome sequencing. BMC Genet. 2017;18(1):14. 10.1186/s12863-017-0479-5. (**Published 2017 Feb 14**). 28193154 10.1186/s12863-017-0479-5 PMC5307692

[17] Lander ES, Linton LM, Birren B, et al. Initial sequencing and analysis of the human genome [published correction appears in Nature 2001 Aug 2;412(6846):565] [published correction appears in Nature 2001;411(6838):720. Szustakowki, J [corrected to Szustakowski, J]]. *Nature*. 2001;409(6822):860–921. 10.1038/35057062 10.1038/35057062 11237011

[18] Tuzun E, Sharp AJ, Bailey JA, et al. Fine-scale structural variation of the human genome. Nat Genet. 2005;37(7):727–32. 10.1038/ng1562. 15895083 10.1038/ng1562

[19] Goodwin S, McPherson JD, McCombie WR. Coming of age: ten years of next-generation sequencing technologies. Nat Rev Genet. 2016;17(6):333–51. 10.1038/nrg.2016.49. 27184599 10.1038/nrg.2016.49 PMC10373632

[20] Majewski J, Schwartzentruber J, Lalonde E, Montpetit A, Jabado N. What can exome sequencing do for you? J Med Genet. 2011;48(9):580–9. 10.1136/jmedgenet-2011-100223. 21730106 10.1136/jmedgenet-2011-100223

[21] Wetterstrand KA. DNA sequencing costs: data from the NHGRI genome sequencing program (GSP). www.genome.gov/sequencingcostsdata. Accessed 11 Jan 2024.

[22] Austin-Tse CA, Jobanputra V, Perry DL, et al. Best practices for the interpretation and reporting of clinical whole genome sequencing. NPJ Genom Med. 2022;7(1):27. 10.1038/s41525-022-00295-z. (**Published 2022 Apr 8**). 35395838 10.1038/s41525-022-00295-z PMC8993917

[23] Pereira R, Oliveira J, Sousa M. Bioinformatics and computational tools for next-generation sequencing analysis in clinical genetics. J Clin Med. 2020;9(1):132. 10.3390/jcm9010132. (**Published 2020 Jan 3**). 31947757 10.3390/jcm9010132 PMC7019349

[24] Zhao Z, Zhang KN, Sun Z, et al. WES data from 286 diffuse gliomas under the 2021 WHO Classification of tumors of the central nervous system. Sci Data. 2022;9(1):692. 10.1038/s41597-022-01823-3. (**Published 2022 Nov 11**). 36369198 10.1038/s41597-022-01823-3 PMC9652316

[25] Nicolas G, Wallon D, Charbonnier C, et al. Screening of dementia genes by whole-exome sequencing in early-onset Alzheimer disease: input and lessons. Eur J Hum Genet. 2016;24(5):710–6. 10.1038/ejhg.2015.173. 26242991 10.1038/ejhg.2015.173 PMC4930083

[26] Yang L, Lee MS, Lu H, et al. Analyzing somatic genome rearrangements in human cancers by using whole-exome sequencing. Am J Hum Genet. 2016;98(5):843–56. 10.1016/j.ajhg.2016.03.017. 27153396 10.1016/j.ajhg.2016.03.017 PMC4863662

[27] Bau S, Schracke N, Kränzle M, et al. Targeted next-generation sequencing by specific capture of multiple genomic loci using low-volume microfluidic DNA arrays. Anal Bioanal Chem. 2009;393(1):171–5. 10.1007/s00216-008-2460-7. 18958448 10.1007/s00216-008-2460-7

[28] Ruiz-Martínez J, Azcona LJ, Bergareche A, Martí-Massó JF, Paisán-Ruiz C. Whole-exome sequencing associates novel *CSMD1* gene mutations with familial Parkinson disease. Neurol Genet. 2017;3(5): e177. 10.1212/NXG.0000000000000177. (**Published 2017 Aug 2**). 28808687 10.1212/NXG.0000000000000177 PMC5540655

[29] Soudyab M, Shariati M, Esfehani RJ, et al. Whole-exome sequencing study of consanguineous parkinson’s disease families and related phenotypes: report of twelve novel variants. J Mol Neurosci. 2022;72(12):2486–96. 10.1007/s12031-022-02085-9. 36520381 10.1007/s12031-022-02085-9

[30] Greisenegger EK, Llufriu S, Chamorro A, et al. A NOTCH3 homozygous nonsense mutation in familial Sneddon syndrome with pediatric stroke. J Neurol. 2021;268(3):810–6. 10.1007/s00415-020-10081-5. 32980981 10.1007/s00415-020-10081-5 PMC7914241

[31] Hao X, Pang J, Li R, et al. Exome sequencing study revealed novel susceptibility loci in subarachnoid hemorrhage (SAH). Mol Brain. 2020;13(1):82. 10.1186/s13041-020-00620-6. (**Published 2020 May 25**). 32450902 10.1186/s13041-020-00620-6 PMC7249693

[32] Håvik AL, Bruland O, Myrseth E, et al. Genetic landscape of sporadic vestibular schwannoma. J Neurosurg. 2018;128(3):911–22. 10.3171/2016.10.JNS161384. 28409725 10.3171/2016.10.JNS161384

[33] Chang LH, Chi NF, Chen CY, et al. Monogenic causes in familial stroke across intracerebral hemorrhage and ischemic stroke subtypes identified by whole-exome sequencing. Cell Mol Neurobiol. 2023;43(6):2769–83. 10.1007/s10571-022-01315-3. 36580209 10.1007/s10571-022-01315-3 PMC10333419

[34] Lee YC, Durr A, Majczenko K, et al. Mutations in KCND3 cause spinocerebellar ataxia type 22. Ann Neurol. 2012;72(6):859–69. 10.1002/ana.23701. 23280837 10.1002/ana.23701 PMC4085146

[35] Carrera C, Jiménez-Conde J, Derdak S, et al. Whole exome sequencing analysis reveals TRPV3 as a risk factor for cardioembolic stroke. Thromb Haemost. 2016;116(6):1165–71. 10.1160/TH16-02-0113. 27604134 10.1160/TH16-02-0113

[36] Williams KL, Warraich ST, Yang S, et al. UBQLN2/ubiquilin 2 mutation and pathology in familial amyotrophic lateral sclerosis. Neurobiol Aging. 2012. 10.1016/j.neurobiolaging.2012.05.008. 22717235 10.1016/j.neurobiolaging.2012.05.008

[37] Holstege H, Hulsman M, Charbonnier C, et al. Exome sequencing identifies rare damaging variants in ATP8B4 and ABCA1 as risk factors for Alzheimer’s disease. Nat Genet. 2022;54(12):1786–94. 10.1038/s41588-022-01208-7. 36411364 10.1038/s41588-022-01208-7 PMC9729101

[38] Clark MJ, Chen R, Lam HY, et al. Performance comparison of exome DNA sequencing technologies. Nat Biotechnol. 2011;29(10):908–14. 10.1038/nbt.1975. (**Published 2011 Sep 25**). 21947028 10.1038/nbt.1975 PMC4127531

[39] Bodi K, Perera AG, Adams PS, et al. Comparison of commercially available target enrichment methods for next-generation sequencing. J Biomol Tech. 2013;24(2):73–86. 10.7171/jbt.13-2402-002. 23814499 10.7171/jbt.13-2402-002 PMC3605921

[40] Chilamakuri CS, Lorenz S, Madoui MA, et al. Performance comparison of four exome capture systems for deep sequencing. BMC Genomics. 2014;15(1):449. 10.1186/1471-2164-15-449. (**Published 2014 Jun 9**). 24912484 10.1186/1471-2164-15-449 PMC4092227

[41] Sulonen AM, Ellonen P, Almusa H, et al. Comparison of solution-based exome capture methods for next generation sequencing. Genome Biol. 2011;12(9):R94. 10.1186/gb-2011-12-9-r94. (**Published 2011 Sep 28**). 21955854 10.1186/gb-2011-12-9-r94 PMC3308057

[42] Asan, Xu Y, Jiang H, et al. Comprehensive comparison of three commercial human whole-exome capture platforms. Genome Biol. 2011;12(9):R95. 10.1186/gb-2011-12-9-r95. 21955857 10.1186/gb-2011-12-9-r95 PMC3308058

[43] Altmüller J, Motameny S, Becker C, et al. A systematic comparison of two new releases of exome sequencing products: the aim of use determines the choice of product. Biol Chem. 2016;397(8):791–801. 10.1515/hsz-2015-0300. 27021259 10.1515/hsz-2015-0300

[44] Raghavan NS, Brickman AM, Andrews H, et al. Whole-exome sequencing in 20,197 persons for rare variants in Alzheimer's disease [published correction appears in Ann Clin Transl Neurol. 2019 Feb 25;6(2):416]. *Ann Clin Transl Neurol*. 2018;5(7):832–842. Published 2018 May 24. 10.1002/acn3.582 10.1002/acn3.582 PMC6043775 30009200

[45] Cukier HN, Kunkle BK, Hamilton KL, et al. Exome sequencing of extended families with Alzheimer’s disease identifies novel genes implicated in cell immunity and neuronal function. J Alzheimers Dis Parkinsonism. 2017;7(4):355. 10.4172/2161-0460.1000355. 29177109 10.4172/2161-0460.1000355 PMC5698805

[46] Vilariño-Güell C, Wider C, Ross OA, et al. VPS35 mutations in Parkinson disease [published correction appears in Am J Hum Genet. 2011 Aug 12;89(2):347]. *Am J Hum Genet*. 2011;89(1):162–167. 10.1016/j.ajhg.2011.06.001 10.1016/j.ajhg.2011.06.001 PMC3135796 21763482

[47] Zimprich A, Benet-Pagès A, Struhal W, et al. A mutation in VPS35, encoding a subunit of the retromer complex, causes late-onset Parkinson disease. Am J Hum Genet. 2011;89(1):168–75. 10.1016/j.ajhg.2011.06.008. 21763483 10.1016/j.ajhg.2011.06.008 PMC3135812

[48] Sandor C, Honti F, Haerty W, et al. Whole-exome sequencing of 228 patients with sporadic Parkinson’s disease. Sci Rep. 2017;7:41188. 10.1038/srep41188. 28117402 10.1038/srep41188 PMC5259721

[49] Pankratz N, Wilk J, Latourelle J, Destefano A, Halter C, Pugh E, Doheny K, Gusella J, Nichols W, Foroud T, Myers R, Investigators C. Genomewide association study for susceptibility genes contributing to familial Parkinson disease. Hum Genet. 2009;124:593–605. 10.1007/s00439-008-0582-9. 18985386 10.1007/s00439-008-0582-9 PMC2627511

[50] Mizuta I, Satake W, Nakabayashi Y, Ito C, Suzuki S, Momose Y, Nagai Y, Oka A, Inoko H, Fukae J, Saito Y, Sawabe M, Murayama S, Yamamoto M, Hattori N, Murata M, Toda T. Multiple candidate gene analysis identifies alpha-synuclein as a susceptibility gene for sporadic Parkinson’s disease. Hum Mol Genet. 2006;15(7):1151–8. 10.1093/HMG/DDL030. 16500997 10.1093/hmg/ddl030

[51] Sebate B, Cuttler K, Cloete R, Britz M, Christoffels A, Williams M, Carr J, Bardien S. Prioritization of candidate genes for a South African family with Parkinson’s disease using in-silico tools. PLoS ONE. 2021. 10.1371/journal.pone.0249324. 33770142 10.1371/journal.pone.0249324 PMC7997022

[52] Zhao Y, Zhang K, Pan H, Wang Y, Zhou X, Xiang Y, Xu Q, Sun Q, Tan J, Yan X, Li J, Guo J, Tang B, Liu Z. Genetic analysis of six transmembrane protein family genes in Parkinson’s disease in a large chinese cohort. Front Aging Neurosci. 2022. 10.3389/fnagi.2022.889057. 35860667 10.3389/fnagi.2022.889057 PMC9289399

[53] Yan W, Tang B, Zhou X, Lei L, Li K, Sun Q, Xu Q, Yan X, Guo J, Liu Z. TMEM230 mutation analysis in Parkinson’s disease in a Chinese population. Neurobiol Aging. 2017;49:219.e1-219.e3. 10.1016/j.neurobiolaging.2016.10.007. 27814995 10.1016/j.neurobiolaging.2016.10.007

[54] Li N, Wang L, Zhang J, et al. Whole-exome sequencing in early-onset Parkinson’s disease among ethnic Chinese. Neurobiol Aging. 2020;90:150.e5-150.e11. 10.1016/j.neurobiolaging.2019.12.023. 32171587 10.1016/j.neurobiolaging.2019.12.023

[55] Johnson JO, Mandrioli J, Benatar M, et al. Exome sequencing reveals VCP mutations as a cause of familial ALS [published correction appears in Neuron. 2011 Jan 27;69(2):397]. *Neuron*. 2010;68(5):857–864. 10.1016/j.neuron.2010.11.036 10.1016/j.neuron.2010.11.036 PMC3032425 21145000

[56] Perlman S. Hereditary Ataxia Overview. 1998 Oct 28 [Updated 2023 Nov 16]. In: Adam MP, Feldman J, Mirzaa GM, et al., editors. GeneReviews®. Seattle (WA): University of Washington, Seattle; 1993–2024. https://www.ncbi.nlm.nih.gov/books/NBK1138/20301317

[57] Novis LE, Alavi S, Pellerin D, et al. Unraveling the genetic landscape of undiagnosed cerebellar ataxia in Brazilian patients. Parkinsonism Relat Disord. 2024;119: 105961. 10.1016/j.parkreldis.2023.105961. 38145611 10.1016/j.parkreldis.2023.105961

[58] Rothblum-Oviatt C, Wright J, Lefton-Greif MA, McGrath-Morrow SA, Crawford TO, Lederman HM. Ataxia telangiectasia: a review. Orphanet J Rare Dis. 2016;11(1):159. 10.1186/s13023-016-0543-7.PMID:27884168;PMCID:PMC5123280. 27884168 10.1186/s13023-016-0543-7 PMC5123280

[59] Bouhouche A, Sefiani S, Charoute H, et al. Novel *WFS1* variants in two Moroccan families with wolfram syndrome. Genet Test Mol Biomarkers. 2024;28(6):257–62. 10.1089/gtmb.2023.0550. 38721948 10.1089/gtmb.2023.0550

[60] Rabbani B, Tekin M, Mahdieh N. The promise of whole-exome sequencing in medical genetics. J Hum Genet. 2014;59(1):5–15. 10.1038/jhg.2013.114. 24196381 10.1038/jhg.2013.114

[61] Sauvigny T, Alawi M, Krause L, et al. Exome sequencing in 38 patients with intracranial aneurysms and subarachnoid hemorrhage. J Neurol. 2020;267(9):2533–45. 10.1007/s00415-020-09865-6. 32367296 10.1007/s00415-020-09865-6 PMC7419486

[62] Farlow JL, Lin H, Sauerbeck L, et al. Lessons learned from whole exome sequencing in multiplex families affected by a complex genetic disorder, intracranial aneurysm. PLoS ONE. 2015;10(3): e0121104. 10.1371/journal.pone.0121104. (**Published 2015 Mar 24**). 25803036 10.1371/journal.pone.0121104 PMC4372548

[63] Ding X, Zhao S, Zhang Q, et al. Exome sequencing reveals a novel variant in *NFX1* causing intracranial aneurysm in a Chinese family. J Neurointerv Surg. 2020;12(2):221–6. 10.1136/neurintsurg-2019-014900. 31401562 10.1136/neurintsurg-2019-014900 PMC7014815

[64] Song Y, Lee JK, Lee JO, Kwon B, Seo EJ, Suh DC. Whole exome sequencing in patients with phenotypically associated familial intracranial aneurysm. Korean J Radiol. 2022;23(1):101–11. 10.3348/kjr.2021.0467. 34668355 10.3348/kjr.2021.0467 PMC8743149

[65] Auer PL, Nalls M, Meschia JF, et al. Rare and coding region genetic variants associated with risk of ischemic stroke: the NHLBI exome sequence project. JAMA Neurol. 2015;72(7):781–8. 10.1001/jamaneurol.2015.0582. 25961151 10.1001/jamaneurol.2015.0582 PMC4673986

[66] Kim DS, Crosslin DR, Auer PL, et al. Rare coding variation in paraoxonase-1 is associated with ischemic stroke in the NHLBI Exome Sequencing Project. J Lipid Res. 2014;55(6):1173–8. 10.1194/jlr.P049247. 24711634 10.1194/jlr.P049247 PMC4031948

[67] Wang K, Zhao S, Zhang Q, et al. Whole-exome sequencing reveals known and novel variants in a cohort of intracranial vertebral-basilar artery dissection (IVAD). J Hum Genet. 2018;63(11):1119–28. 10.1038/s10038-018-0496-x. 30115950 10.1038/s10038-018-0496-x

[68] Mönkäre S, Kuuluvainen L, Kun-Rodrigues C, et al. Whole-exome sequencing of Finnish patients with vascular cognitive impairment. Eur J Hum Genet. 2021;29(4):663–71. 10.1038/s41431-020-00775-9. 33268848 10.1038/s41431-020-00775-9 PMC8115269

[69] Yılmaz B, Toktaş ZO, Akakın A, et al. Familial occurrence of brain arteriovenous malformation: a novel ACVRL1 mutation detected by whole exome sequencing. J Neurosurg. 2017;126(6):1879–83. 10.3171/2016.6.JNS16665. 27611203 10.3171/2016.6.JNS16665

[70] Zhang M, Ding X, Zhang Q, et al. Exome sequencing of 112 trios identifies recessive genetic variants in brain arteriovenous malformations. J Neurointerv Surg. 2021;13(6):568–73. 10.1136/neurintsurg-2020-016469. 32848021 10.1136/neurintsurg-2020-016469

[71] Alexandrov LB, Nik-Zainal S, Wedge DC, Aparicio SA, Behjati S, Biankin AV, Bignell GR, Bolli N, Borg A, Børresen-Dale AL, Boyault S, Burkhardt B, Butler AP, Caldas C, Davies HR, Desmedt C, Eils R, Eyfjörd JE, Foekens JA, Greaves M, Hosoda F, Hutter B, Ilicic T, Imbeaud S, Imielinski M, Jäger N, Jones DT, Jones D, Knappskog S, Kool M, Lakhani SR, López-Otín C, Martin S, Munshi NC, Nakamura H, Northcott PA, Pajic M, Papaemmanuil E, Paradiso A, Pearson JV, Puente XS, Raine K, Ramakrishna M, Richardson AL, Richter J, Rosenstiel P, Schlesner M, Schumacher TN, Span PN, Teague JW, Totoki Y, Tutt AN, Valdés-Mas R, van Buuren MM, van 't Veer L, Vincent-Salomon A, Waddell N, Yates LR; Australian Pancreatic Cancer Genome Initiative; ICGC Breast Cancer Consortium; ICGC MMML-Seq Consortium; ICGC PedBrain; Zucman-Rossi J, Futreal PA, McDermott U, Lichter P, Meyerson M, Grimmond SM, Siebert R, Campo E, Shibata T, Pfister SM, Campbell PJ, Stratton MR. Signatures of mutational processes in human cancer. Nature. 2013;500(7463):415–21. 10.1038/nature12477. Epub 2013 Aug 14. Erratum in: Nature. 2013;502(7470):258. Imielinsk, Marcin [corrected to Imielinski, Marcin]. 10.1038/nature12477 PMC3776390 23945592

[72] Rahman N. Realizing the promise of cancer predisposition genes. Nature. 2014;505(7483):302-8. 10.1038/nature12981. Erratum in: Nature. 2014;510(7503):176. 10.1038/nature12981 PMC4975511 24429628

[73] Robson ME, Bradbury AR, Arun B, Domchek SM, Ford JM, Hampel HL, Lipkin SM, Syngal S, Wollins DS, Lindor NM. American society of clinical oncology policy statement update: genetic and genomic testing for cancer susceptibility. J Clin Oncol. 2015;33(31):3660–7. 10.1200/JCO.2015.63.0996. (**Epub 2015 Aug 31**). 26324357 10.1200/JCO.2015.63.0996

[74] Vaubel RA, Tian S, Remonde D, et al. Genomic and phenotypic characterization of a broad panel of patient-derived xenografts reflects the diversity of glioblastoma. Clin Cancer Res. 2020;26(5):1094–104. 10.1158/1078-0432.CCR-19-0909. 31852831 10.1158/1078-0432.CCR-19-0909 PMC7056576

[75] Shi ZF, Li KK, Kwan JSH, et al. Whole-exome sequencing revealed mutational profiles of giant cell glioblastomas. Brain Pathol. 2019;29(6):782–92. 10.1111/bpa.12720. 30861589 10.1111/bpa.12720 PMC8028679

[76] Rajappa P, Eng KW, Bareja R, et al. Utility of multimodality molecular profiling for pediatric patients with central nervous system tumors. Neurooncol Adv. 2022;4(1):vdac031. 10.1093/noajnl/vdac031. (**Published 2022 Mar 10**). 35475276 10.1093/noajnl/vdac031 PMC9034114

[77] Sun J, Wang C, Zhang Y, et al. Genomic signatures reveal DNA damage response deficiency in colorectal cancer brain metastases. Nat Commun. 2019;10(1):3190. 10.1038/s41467-019-10987-3. (**Published 2019 Jul 18**). 31320627 10.1038/s41467-019-10987-3 PMC6639368

[78] Routh ED, Van Swearingen AED, Sambade MJ, et al. Comprehensive analysis of the immunogenomics of triple-negative breast cancer brain metastases from LCCC1419. Front Oncol. 2022;12: 818693. 10.3389/fonc.2022.818693. (**Published 2022 Jul 27**). 35992833 10.3389/fonc.2022.818693 PMC9387304

[79] Liu Z, Zheng M, Lei B, et al. Whole-exome sequencing identifies somatic mutations associated with lung cancer metastasis to the brain. Ann Transl Med. 2021;9(8):694. 10.21037/atm-21-1555. 33987392 10.21037/atm-21-1555 PMC8106079

[80] Yu J, Lai M, Zhou Z, et al. The PTEN-associated immune prognostic signature reveals the landscape of the tumor microenvironment in glioblastoma. J Neuroimmunol. 2023;376: 578034. 10.1016/j.jneuroim.2023.578034. 36791582 10.1016/j.jneuroim.2023.578034

[81] Murakami K, Kikugawa S, Seki S, et al. Exome sequencing reveals De Novo variants in congenital scoliosis. J Pediatr Genet. 2021;11(4):287–91. 10.1055/s-0041-1726282. 36267860 10.1055/s-0041-1726282 PMC9578779

[82] Li Z, Zhao S, Cai S, et al. The mutational burden and oligogenic inheritance in Klippel-Feil syndrome. BMC Musculoskelet Disord. 2020;21(1):220. 10.1186/s12891-020-03229-x. 32278351 10.1186/s12891-020-03229-x PMC7149842

[83] Takeda K, Kou I, Mizumoto S, et al. Screening of known disease genes in congenital scoliosis. Mol Genet Genomic Med. 2018;6(6):966–74. 10.1002/mgg3.466. 30196550 10.1002/mgg3.466 PMC6305645

[84] Lee CH, Kim KT, Kim CH, et al. Unveiling the genetic variation of severe continuous/mixed-type ossification of the posterior longitudinal ligament by whole-exome sequencing and bioinformatic analysis. Spine J. 2021;21(11):1847–56. 10.1016/j.spinee.2021.07.005. 34273568 10.1016/j.spinee.2021.07.005

[85] Mierzewska H, Rydzanicz M, Biegański T, et al. Spondyloepimetaphyseal dysplasia with neurodegeneration associated with AIFM1 mutation—a novel phenotype of the mitochondrial disease. Clin Genet. 2017;91(1):30–7. 10.1111/cge.12792. 27102849 10.1111/cge.12792

[86] Wang C, Seltzsam S, Zheng B, et al. Whole exome sequencing identifies potential candidate genes for spina bifida derived from mouse models. Am J Med Genet A. 2022;188(5):1355–67. 10.1002/ajmg.a.62644. 35040250 10.1002/ajmg.a.62644 PMC8995376

[87] Chang HR, Cho SY, Lee JH, et al. Hypomorphic mutations in TONSL cause SPONASTRIME dysplasia. Am J Hum Genet. 2019;104(3):439–53. 10.1016/j.ajhg.2019.01.009. 30773278 10.1016/j.ajhg.2019.01.009 PMC6407524

[88] Jiang X, Chen D. The identification of novel gene mutations for degenerative lumbar spinal stenosis using whole-exome sequencing in a Chinese cohort. BMC Med Genomics. 2021;14:134. 10.1186/s12920-021-00981-4. 34020649 10.1186/s12920-021-00981-4 PMC8138972

[89] Terhune EA, Wethey CI, Cuevas MT, et al. Whole exome sequencing of 23 multigeneration idiopathic scoliosis families reveals enrichments in cytoskeletal variants, suggests highly polygenic disease. Genes (Basel). 2021;12(6):922. 10.3390/genes12060922. (**Published 2021 Jun 16**). 34208743 10.3390/genes12060922 PMC8235452

[90] Terhune EA, Cuevas MT, Monley AM, et al. Mutations in KIF7 implicated in idiopathic scoliosis in humans and axial curvatures in zebrafish. Hum Mutat. 2021;42(4):392–407. 10.1002/humu.24162. 33382518 10.1002/humu.24162 PMC8049985

[91] Fisher RS, Acevedo C, Arzimanoglou A, et al. ILAE official report: a practical clinical definition of epilepsy. Epilepsia. 2014;55(4):475–82. 10.1111/epi.12550. 24730690 10.1111/epi.12550

[92] Zhang L, Gao J, Liu H, et al. Pathogenic variants identified by whole-exome sequencing in 43 patients with epilepsy. Hum Genomics. 2020;14(1):44. 10.1186/s40246-020-00294-0. (**Published 2020 Dec 7**). 33287870 10.1186/s40246-020-00294-0 PMC7720389

[93] Demos M, Guella I, DeGuzman C, et al. Diagnostic yield and treatment impact of targeted exome sequencing in early-onset epilepsy. Front Neurol. 2019;10:434. 10.3389/fneur.2019.00434. (**Published 2019 May 21**). 31164858 10.3389/fneur.2019.00434 PMC6536592

[94] Rochtus A, Olson HE, Smith L, et al. Genetic diagnoses in epilepsy: the impact of dynamic exome analysis in a pediatric cohort. Epilepsia. 2020;61(2):249–58. 10.1111/epi.16427. 31957018 10.1111/epi.16427 PMC7404709

[95] Bi W, Glass IA, Muzny DM, et al. Whole exome sequencing identifies the first STRADA point mutation in a patient with polyhydramnios, megalencephaly, and symptomatic epilepsy syndrome (PMSE). Am J Med Genet A. 2016;170(8):2181–5. 10.1002/ajmg.a.37727. 27170158 10.1002/ajmg.a.37727

[96] Dunn PJ, Maher BH, Albury CL, et al. Tiered analysis of whole-exome sequencing for epilepsy diagnosis. Mol Genet Genomics. 2020;295(3):751–63. 10.1007/s00438-020-01657-x. 32146541 10.1007/s00438-020-01657-x

[97] Naseer MI, Abdulkareem AA, Rasool M, Algahtani H, Muthaffar OY, Pushparaj PN. Whole-exome sequencing identifies novel *SCN1A* and *CACNB4* genes mutations in the cohort of saudi patients with epilepsy. Front Pediatr. 2022;10: 919996. 10.3389/fped.2022.919996. (**Published 2022 Jun 22**). 35813387 10.3389/fped.2022.919996 PMC9257097

[98] Zeng B, Zhang H, Lu Q, et al. Identification of five novel *SCN1A* variants. Front Behav Neurosci. 2023;17:1272748. 10.3389/fnbeh.2023.1272748. 38025388 10.3389/fnbeh.2023.1272748 PMC10663289

[99] Du S, Zeng S, Song L, et al. Functional characterization of novel NPRL3 mutations identified in three families with focal epilepsy. Sci China Life Sci. 2023;66(9):2152–66. 10.1007/s11427-022-2313-1. 37071290 10.1007/s11427-022-2313-1

[100] Zhang H, Deng J, Wang X, et al. Clinical phenotypic and genotypic characterization of *NPRL3*-related epilepsy. Front Neurol. 2023;14:1113747. 10.3389/fneur.2023.1113747. (**Published 2023 Mar 2**). 36937533 10.3389/fneur.2023.1113747 PMC10018541

[101] Chuan Z, Ruikun C, Qian L, et al. Genetic and phenotype analysis of a Chinese cohort of infants and children with epilepsy. Front Genet. 2022;13: 869210. 10.3389/fgene.2022.869210. (**Published 2022 Apr 27**). 35571021 10.3389/fgene.2022.869210 PMC9091957

[102] Lee S, Karp N, Zapata-Aldana E, et al. Genetic testing in children with epilepsy: report of a single-center experience. Can J Neurol Sci. 2021;48(2):233–44. 10.1017/cjn.2020.167. 32741404 10.1017/cjn.2020.167

[103] Costa C, Oliver KL, Calvello C, et al. IRF2BPL: a new genotype for progressive myoclonus epilepsies. Epilepsia. 2023;64(8):e164–9. 10.1111/epi.17557. 36810721 10.1111/epi.17557

[104] Li S, Yu S, Zhang Y, Wang Y, Jiang X, Wu C. Compound heterozygous loss-of-function variants in BRAT1 cause lethal neonatal rigidity and multifocal seizure syndrome. Mol Genet Genomic Med. 2023;11(1): e2092. 10.1002/mgg3.2092. 36367347 10.1002/mgg3.2092 PMC9834191

[105] Leitner DF, Lin Z, Sawaged Z, et al. Brain molecular mechanisms in Rasmussen encephalitis. Epilepsia. 2023;64(1):218–30. 10.1111/epi.17457. 36336987 10.1111/epi.17457 PMC9852002

[106] Choi M, Scholl UI, Ji W, et al. Genetic diagnosis by whole exome capture and massively parallel DNA sequencing. Proc Natl Acad Sci U S A. 2009;106(45):19096–101. 10.1073/pnas.0910672106. 19861545 10.1073/pnas.0910672106 PMC2768590

[107] Monroe GR, Frederix GW, Savelberg SM, et al. Effectiveness of whole-exome sequencing and costs of the traditional diagnostic trajectory in children with intellectual disability. Genet Med. 2016;18(9):949–56. 10.1038/gim.2015.200. 26845106 10.1038/gim.2015.200

[108] Ewans LJ, Schofield D, Shrestha R, et al. Whole-exome sequencing reanalysis at 12 months boosts diagnosis and is cost-effective when applied early in Mendelian disorders. Genet Med. 2018;20(12):1564–74. 10.1038/gim.2018.39. 29595814 10.1038/gim.2018.39

[109] Westra D, Schouten MI, Stunnenberg BC, et al. Panel-based exome sequencing for neuromuscular disorders as a diagnostic service. J Neuromuscul Dis. 2019;6(2):241–58. 10.3233/JND-180376. 31127727 10.3233/JND-180376

[110] Ewans LJ, et al. Whole exome and genome sequencing in mendelian disorders: a diagnostic and health economic analysis. Eur J Human Genet EJHG. 2022;30(10):1121–31. 10.1038/s41431-022-01162-2. 35970915 10.1038/s41431-022-01162-2 PMC9553973

[111] Bick D, Dimmock D. Whole exome and whole genome sequencing. Curr Opin Pediatr. 2011;23(6):594–600. 10.1097/MOP.0b013e32834b20ec. 21881504 10.1097/MOP.0b013e32834b20ec

[112] Richards S, et al. Standards and guidelines for the interpretation of sequence variants: a joint consensus recommendation of the American College of Medical Genetics and Genomics and the Association for Molecular Pathology. Genet Med. 2015;17(5):405–24. 10.1038/gim.2015.30. 25741868 10.1038/gim.2015.30 PMC4544753

[113] Xue Y, Ankala A, Wilcox WR, Hegde MR. Solving the molecular diagnostic testing conundrum for Mendelian disorders in the era of next-generation sequencing: single-gene, gene panel, or exome/genome sequencing. Genet Med. 2015;17(6):444–51. 10.1038/gim.2014.122. 25232854 10.1038/gim.2014.122

[114] Ream MA, Mikati MA. Clinical utility of genetic testing in pediatric drug-resistant epilepsy: a pilot study. Epilepsy Behav. 2014;37:241–8. 10.1016/j.yebeh.2014.06.018. 25108116 10.1016/j.yebeh.2014.06.018

[115] Newman WG, Black GC. Delivery of a clinical genomics service. Genes (Basel). 2014;5(4):1001–17. 10.3390/genes5041001. (**Published 2014 Nov 6**). 25383561 10.3390/genes5041001 PMC4276923

[116] Lazaridis KN, McAllister TM, Babovic-Vuksanovic D, et al. Implementing individualized medicine into the medical practice. Am J Med Genet C Semin Med Genet. 2014;166C(1):15–23. 10.1002/ajmg.c.31387. 24616301 10.1002/ajmg.c.31387

[117] Kamalakaran S, Varadan V, Janevski A, et al. Translating next generation sequencing to practice: opportunities and necessary steps. Mol Oncol. 2013;7(4):743–55. 10.1016/j.molonc.2013.04.008. 23769412 10.1016/j.molonc.2013.04.008 PMC5528427

[118] Krabbenborg L, Vissers LE, Schieving J, et al. Understanding the psychosocial effects of WES test results on parents of children with rare diseases. J Genet Couns. 2016;25(6):1207–14. 10.1007/s10897-016-9958-5. 27098417 10.1007/s10897-016-9958-5 PMC5114322

[119] Jurgens J, Ling H, Hetrick K, et al. Assessment of incidental findings in 232 whole-exome sequences from the Baylor-Hopkins Center for Mendelian Genomics. Genet Med. 2015;17(10):782–8. 10.1038/gim.2014.196. 25569433 10.1038/gim.2014.196 PMC4496331

[120] Hallowell N, Hall A, Alberg C, Zimmern R. Revealing the results of whole-genome sequencing and whole-exome sequencing in research and clinical investigations: some ethical issues. J Med Ethics. 2015;41(4):317–21. 10.1136/medethics-2013-101996. 25038088 10.1136/medethics-2013-101996

[121] Tabor HK, Berkman BE, Hull SC, Bamshad MJ. Genomics really gets personal: how exome and whole genome sequencing challenge the ethical framework of human genetics research. Am J Med Genet A. 2011;155A(12):2916–24. 10.1002/ajmg.a.34357. 22038764 10.1002/ajmg.a.34357 PMC4819320

[122] Martinez-Martin N, Magnus D. Privacy and ethical challenges in next-generation sequencing. Expert Rev Precis Med Drug Dev. 2019;4(2):95–104. 10.1080/23808993.2019.1599685. 32775691 10.1080/23808993.2019.1599685 PMC7413244

[123] Klein H, Bauer P, Hambuch T. Whole genome sequencing (WGS), whole exome sequencing (WES) and clinical exome sequencing (CES) in patient care. LaboratoriumsMedizin. 2014;38(4):221–30. 10.1515/labmed-2014-0025.

[124] Alvarez-Mora MI, Rodríguez-Revenga L, Jodar M, et al. Implementation of exome sequencing in clinical practice for neurological disorders. Genes (Basel). 2023;14(4):813. 10.3390/genes14040813. 37107571 10.3390/genes14040813 PMC10137364

[125] Ferraro NM, Strober BJ, Einson J, et al. Transcriptomic signatures across human tissues identify functional rare genetic variation. Science. 2020;369(6509):eaaz5900. 10.1126/science.aaz5900. 32913073 10.1126/science.aaz5900 PMC7646251

[126] Shah SH, Newgard CB. Integrated metabolomics and genomics: systems approaches to biomarkers and mechanisms of cardiovascular disease. Circ Cardiovasc Genet. 2015;8(2):410–9. 10.1161/CIRCGENETICS.114.000223. 25901039 10.1161/CIRCGENETICS.114.000223 PMC4408557

[127] Crowther LM, Poms M, Plecko B. Multiomics tools for the diagnosis and treatment of rare neurological disease. J Inherit Metab Dis. 2018;41(3):425–34. 10.1007/s10545-018-0154-7. 29536202 10.1007/s10545-018-0154-7 PMC5959950

[128] Suwinski P, Ong C, Ling MHT, Poh YM, Khan AM, Ong HS. Advancing personalized medicine through the application of whole exome sequencing and big data analytics. Front Genet. 2019;10:49. 10.3389/fgene.2019.00049. (**Published 2019 Feb 12**). 30809243 10.3389/fgene.2019.00049 PMC6379253

[129] Khoja L, Butler MO, Kang SP, Ebbinghaus S, Joshua AM. Pembrolizumab. J Immunother Cancer. 2015;3:36. 10.1186/s40425-015-0078-9. (**Published 2015 Aug 18**). 26288737 10.1186/s40425-015-0078-9 PMC4539882

[130] Yang T, et al. PD-1/PD-L1 immune checkpoint inhibitors in glioblastoma: clinical studies, challenges and potential. Human Vaccines Immunother. 2021;17(2):546–53. 10.1080/21645515.2020.1782692. 10.1080/21645515.2020.1782692 PMC7899692 32643507

[131] Rexach J, Lee H, Martinez-Agosto JA, Németh AH, Fogel BL. Clinical application of next-generation sequencing to the practice of neurology. Lancet Neurol. 2019;18(5):492–503. 10.1016/S1474-4422(19)30033-X. 30981321 10.1016/S1474-4422(19)30033-X PMC7055532

[132] Harrison R. Whole exome sequencing predicts whether patients respond to cancer immunotherapy. www.nyu.edu/about/news-publications/news/2022/july/whole-exome-sequencing-cancer-immunotherapy.html. Accessed 11 Jan 2024.

[133] Tarailo-Graovac M, Shyr C, Ross CJ, et al. Exome Sequencing and the Management of Neurometabolic Disorders. N Engl J Med. 2016;374(23):2246–55. 10.1056/NEJMoa1515792. 27276562 10.1056/NEJMoa1515792 PMC4983272

[134] Fogel BL, Satya-Murti S, Cohen BH. Clinical exome sequencing in neurologic disease [published correction appears in Neurol Clin Pract. 2016;6(4):368]. *Neurol Clin Pract*. 2016;6(2):164–176. 10.1212/CPJ.0000000000000239 10.1212/CPJ.0000000000000239 PMC4828678 27104068

[135] McInnes G, Sharo AG, Koleske ML, et al. Opportunities and challenges for the computational interpretation of rare variation in clinically important genes. Am J Hum Genet. 2021;108(4):535–48. 10.1016/j.ajhg.2021.03.003. 33798442 10.1016/j.ajhg.2021.03.003 PMC8059338

[136] Liu X, Wu C, Li C, Boerwinkle E. dbNSFP v3.0: a one-stop database of functional predictions and annotations for human nonsynonymous and splice-site SNVs. Hum Mutat. 2016;37(3):235–41. 10.1002/humu.22932. 26555599 10.1002/humu.22932 PMC4752381

[137] Prokop JW, May T, Strong K, et al. Genome sequencing in the clinic: the past, present, and future of genomic medicine. Physiol Genomics. 2018;50(8):563–79. 10.1152/physiolgenomics.00046.2018. 29727589 10.1152/physiolgenomics.00046.2018 PMC6139636

